# Triazole-(*p*-tolylthio)methyl hybrids *via* click chemistry: synthesis, molecular docking, and evaluation as promising anticancer candidates

**DOI:** 10.1039/d5ra09528j

**Published:** 2026-01-20

**Authors:** Tamer El Malah, Ahmed A. El-Rashedy, Randa E. Abdel-Mageid, Aymn E. Rashad, Hanan A. Soliman, Hanem M. Awad, Ahmed H. Shamroukh

**Affiliations:** a Photochemistry Department, Chemical Industries Research Institute, National Research Centre 33 El Buhouth Street, P.O. Box 12622 Cairo Egypt tmara_nrc3000@yahoo.com; b Department of Organic and Medicinal Chemistry, Faculty of Pharmacy, University of Sadat City Menoufia 32897 Egypt; c Chemistry of Natural and Microbial Products Department, National Research Centre Dokki 12622 Cairo Egypt; d Department of Tanning Materials and Leather Technology, National Research Centre 33 El Buhouth Street, P.O. Box 12311 Cairo Egypt

## Abstract

A series of novel (*p*-tolylthio)methyl-linked 1,2,3-triazole derivatives 10–17 was synthesized *via* Cu(i)-catalyzed click chemistry and structurally characterized. The prepared compounds were evaluated for cytotoxicity against HCT-116, HepG2, and MCF-7 cancer cell lines, with BJ-1 fibroblasts as a control. Several derivatives, notably compounds 13 and 17, showed strong activity against HepG2 and MCF-7 cells (IC_50_ ≈ 1.4–1.6 µM), exceeding the activity reference drug doxorubicin, while maintaining low toxicity toward normal cells. Molecular docking studies proposed a potential interaction with the EGFR kinase active site, proposing a hypothetical mechanism that requires further experimental validation. Notably, derivatives 13 and 17 formed additional hydrogen bonds with key residues (Lys745, Leu799, Asp800), suggesting enhanced binding stability and inhibitory potential. These findings identify (*p*-tolylthio)methyl-triazole hybrids as promising anticancer candidates worthy of further development.

## Abstract 2

A series of novel (*p*-tolylthio)methyl-linked 1,2,3-triazole derivatives 10–17 was synthesized *via* Cu(i)-catalyzed click chemistry and structurally characterized.

## Graphical abstract

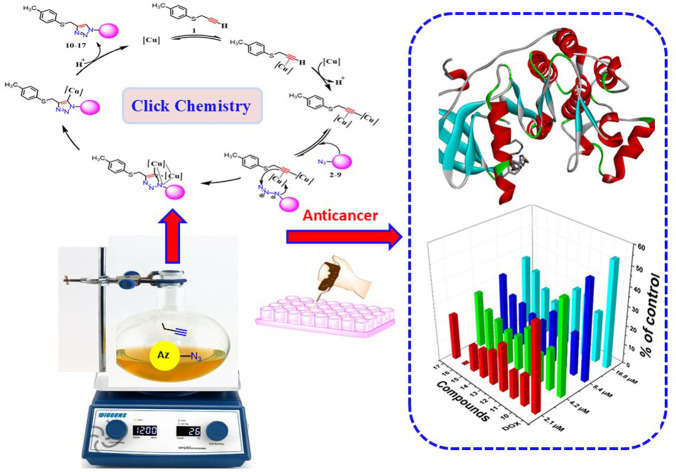

## 1 Introduction

**Fig. 1:**
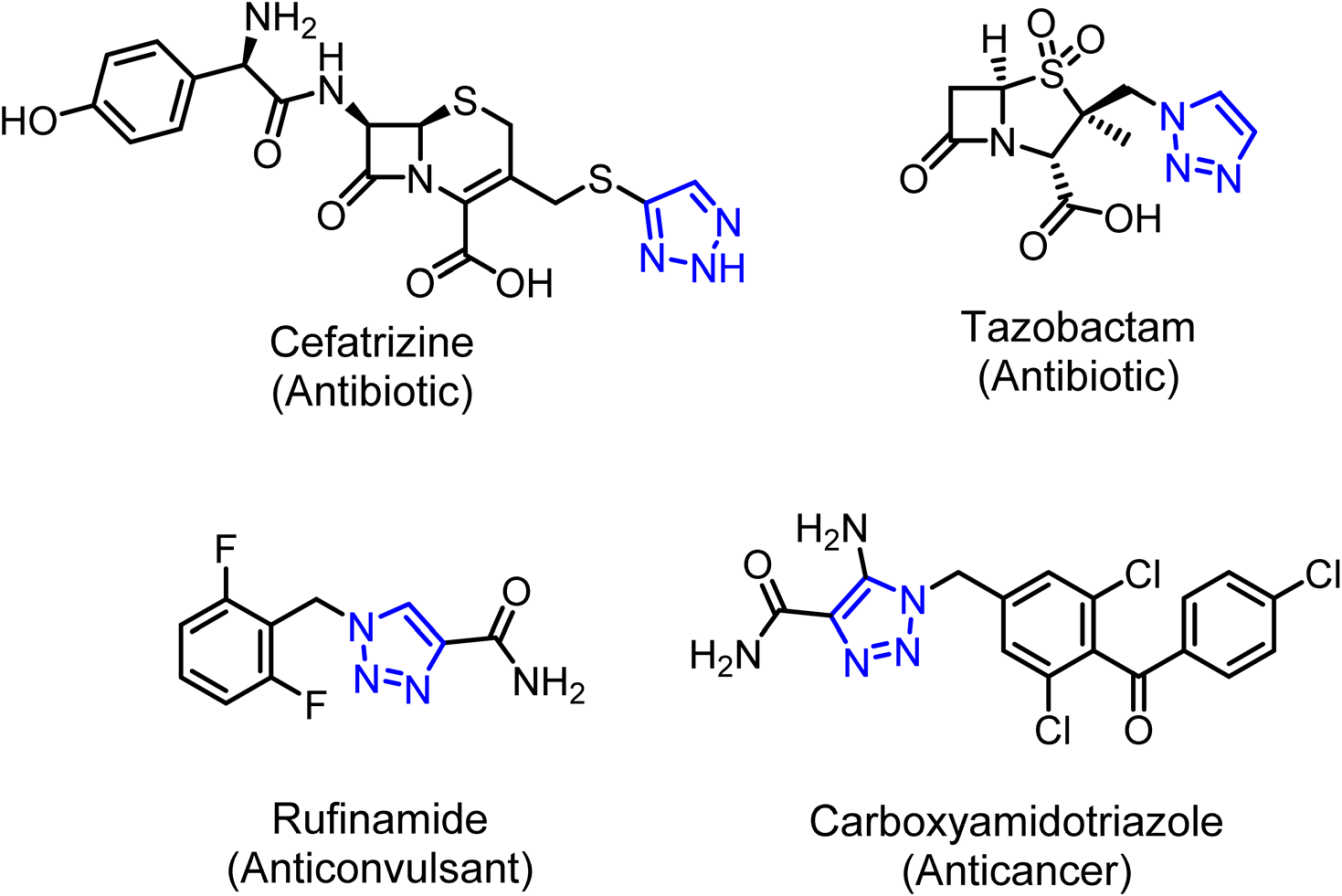
Chemical structures of some clinical drugs having 1,2,3-triazole moieties.

Cancer remains one of the most pressing global health challenges, responsible for millions of deaths annually and placing an enormous burden on patients, families, and healthcare systems.^1^ There are multiple disorders associated with cancer, including uncontrolled cell growth, invasion of surrounding tissues by abnormal cells, and the potential for metastasis.^2^ Unlike normal cells, which grow and die in a regulated manner, cancer cells bypass these controls, leading to malignant tumor formation and disruption of vital bodily functions.^3^ The origins of cancer are multifactorial, involving genetic mutations, environmental exposure, and lifestyle-related risks.^4^ Despite remarkable advances in surgery, chemotherapy, radiotherapy, immunotherapy, and targeted therapies, cancer continues to be a leading cause of mortality worldwide.^5^ In recent years, increasing attention has been directed toward naturally occurring compounds with anticancer potential, particularly sulfur-containing compounds.^6^ These bioactive molecules, found abundantly in foods such as garlic, onions, and cruciferous vegetables, as well as in endogenous molecules like glutathione, play critical roles in cancer prevention and treatment.^7^ Sulfur compounds act through multiple mechanisms: they neutralize carcinogens and reactive oxygen species, enhance detoxification pathways, regulate gene expression, and induce programmed cell death (apoptosis) selectively in cancer cells.^8^ Moreover, they exhibit strong anti-inflammatory and anti-proliferative properties by modulating key signaling pathways involved in tumor growth. Importantly, sulfur compounds also enhance the effectiveness of conventional therapies while protecting normal tissues from treatment-induced damage.^9^ Thus, the study of sulfur compounds in the context of cancer is of great importance.^10^ They not only contribute to a deeper understanding of cancer biology but also offer promising avenues for the development of safer, more effective strategies for both cancer prevention and therapy. Moreover, compounds containing the (*p*-tolylthio)methyl moiety embody a unique combination of lipophilicity and chemical reactivity, which renders it a valuable fragment in both biological and industrial chemistry.^11^ Derivatives containing the arylthioether scaffold, such as alkyl or acyl thioesters, frequently exhibit significant antimicrobial, antioxidant, and cytotoxic properties.^12^ For instance, a series of alkyl 2-(acylthio)benzoates demonstrated pronounced phytogrowth inhibitory and cytotoxic activities, particularly in derivatives featuring acetylthio and propionylthio substituents.^13^ Additionally, compounds like *N*-(benzo[*d*][1,3]dioxol-5-yl)thiazol-2-yl-4-(*p*-tolylthio)butanamide have shown antimicrobial,^14^ anticancer, and antimalarial activities with low micromolar to subnanomolar potency.^15^ Similarly, *N*-(benzo[*d*]thiazol-6-yl)-2-(*p*-tolylthio)acetamide exhibited COX-2 inhibition in the low micromolar range (*e.g.*, IC_50_ ≈ 0.5 µM) and anticancer activity against breast, colon, and cervical cancer cell lines.^16^ The presence of the thioether linkage, particularly one connected to an aromatic ring, often enhances membrane permeability, and sulfur-centered fragments are susceptible to oxidative metabolism into sulfoxides or sulfones—modifications that can modulate pharmacodynamics or serve as prodrug strategies.^17^ From a synthetic chemistry perspective, arylthioether moieties—including (*p*-tolylthio)methyl fragments—play vital roles as protecting groups, synthetic intermediates, and building blocks in complex molecule synthesis.^18^ It has been shown that aromatic thioethers can protect thiols under Suzuki–Miyaura coupling conditions by allowing chemoselective cross-coupling without masking reactive thiol functionality.^19^ Thioether-substituted aryl carbonate groups, such as 4-methylthio-aryl carbonate, have been incorporated as 5′-protecting groups in thymidine phosphoramidites, offering controlled cleavage *via* oxidative conditions (*e.g.*, with H_2_O_2_) during DNA/RNA synthesis.^20^ The overarching importance of protecting groups in multi-step organic synthesis is well-established—their selection is essential for chemoselectivity, and (*p*-tolylthio)methyl-type functionalities offer valuable stability and deprotection profiles.^21^ On the other hand, there are several ways for the synthesis of 1,2,3-triazoles in click chemistry; however, the Cu(i)-catalyzed Huisgen 1,3-dipolar cycloaddition (CuAAC) method is the most popular, in which azides are treated with different alkynes to produce 1,4-regioisomeric triazoles.^22^ Moreover, 1,2,3-triazoles have been reported to exhibit antimicrobial,^23^ anticancer,^24^ antiviral,^25^ antidiabetic,^26^ anti-inflammatory,^27^ anti-Alzheimer's,^28^ and antioxidant activities.^29^ Furthermore, 1,2,3-triazoles have gained interest from materials scientists, especially for their applications in self-assembling materials,^30^ and they are found in the structures of many drugs, *e.g.*, cefatrizine, tazobactam, rufinamide, and carboxyamidotriazole (Fig. 1). As a result of the research mentioned above, the combination of (*p*-tolylthio)methyl and 1,2,3-triazole moieties will be fascinating. Therefore, the current study aims to prepare a series of (*p*-tolylthio)methyl derivatives linked to a 1,2,3-substituted triazole moiety through click chemistry, in an attempt to obtain new compounds that have significant anticancer activity.

## 2 Experimental

### 2.1 Chemistry

#### 2.1.1 Materials and methods

All chemicals were purchased from Sigma-Aldrich and were used as obtained without further purification. The progress of the chemical reactions was monitored by thin-layer chromatography (TLC). TLC was performed on aluminum sheets, TLC silica gel 60 F 254 (20 × 20 cm). The products were purified by column chromatography (silica gel 60, 0.040–0.063 mm) under flash conditions. ^1^H and ^13^C NMR spectra were recorded on a Bruker High-Performance Digital FT NMR spectrometer, Avance III (400 MHz for ^1^H and 100 MHz for 13C NMR). ^1^H and ^13^C NMR signals were referenced to tetramethylsilane (TMS) and the solvent shifts of CDCl_3_. The abbreviations used in reporting ^1^H NMR data were denoted as follows: s, singlet; brs, broad singlet; d, doublet; t, triplet; m, multiplet. The coupling constant values (*J*) were recorded in hertz (Hz). Electron impact mass spectra were measured using a DI Analysis Shimadzu QP-2010 plus (70 eV). Elemental analyses were performed using a CHNS-932 (LECO) Vario Elemental Analyzer.

#### 2.1.2 General procedure for the synthesis of 4-((*p*-tolylthio)methyl)-1*H*-1,2,3-triazole derivatives (10–17)

A three-necked flask was charged with prop-2-yn-1-yl(*p*-tolyl)sulfane 1 (1 equiv.), azide derivatives 2–9 (1 equiv.), sodium ascorbate (0.3 equiv.), TBTA (0.15 equiv.) and a solvent mixture of H_2_O/^*t*^BuOH/CH_2_Cl_2_ (1/2/8, 30 mL). The flask was evacuated and flushed with argon repeatedly (three cycles). Thereafter, CuSO_4_·5H_2_O was added (0.15 equiv.) and the mixture was stirred for 2 days at r.t. in dark. After consumption of alkyne 1 as indicated by TLC monitoring, the mixture was diluted with CH_2_Cl_2_ and transferred into a separating funnel. The organic phase was separated and washed with an aqueous solution of ethylenediaminetetraacetic acid (EDTA) disodium salt (EDTA-Na_2_) (3×). The aqueous phase was extracted with CH_2_Cl_2_ (3×) and washed with a saturated aqueous solution of NaCl (60 mL volume). The organic phase was dried over anhydrous MgSO_4_ and filtered. The solvent was removed under vacuo and the target compound was obtained after purification with column chromatography.

##### 2.1.2.1 1-(4-Iodophenyl)-4-((*p*-tolylthio)methyl)-1*H*-1,2,3-triazole (10)

The title compound as a brown solid (89%). TLC (petroleum ether : ethyl acetate 8/2) *R*_f_ = 0.40. ^1^H-NMR (400 MHz, CDCl_3_): *δ* (ppm) = 2.21 (s, 3H, C*H*_3_), 4.16 (s, 2H, SC*H*_2_), 6.99 (d, *J* = 7.92 Hz, 2H, Ar*H*), 7.16 (d, *J* = 8.09 Hz, 2H, Ar*H*), 7.31 (d, *J* = 8.72 Hz, 2H, Ar*H*), 7.66 (s, 1H, Ar*H*_triazole_), 7.69 (d, *J* = 8.70 Hz, 2H, Ar*H*). ^13^C-NMR (100 MHz, CDCl_3_): *δ* (ppm) = 21.08 (*C*H_3_), 29.44 (S*C*H_2_), 93.58 (I-*C*_Ar_), 120.03 (*C*_Ar_), 121.91 (*C*_Ar_), 129.89 (*C*_Ar_), 130.43 (*C*_Ar_), 131.46 (*C*_Ar_), 134.06 (*C*_Ar_), 136.60 (*C*_Ar_), 136.94 (CH_3_*C*_Ar_), 138.80 (*C*_Ar_). MS (EI, 70 eV): *m*/*z* = 407.32, [M^+^]. Elemental analysis, calcd (%) for C_16_H_14_IN_3_S: *C* 47.19, *H* 3.46, *N* 10.32, *S* 7.87; found *C* 47.37, *H* 3.28, *N* 10.48, *S* 8.12.

##### 2.1.2.2 1-(2,5-Dimethoxy-4-nitrophenyl)-4-((*p*-tolylthio)methyl)-1*H*-1,2,3-triazole (11)

The title compound as a brown solid (92%). TLC (petroleum ether : ethyl acetate 8/2) *R*_f_ = 0.34. ^1^H-NMR (400 MHz, CDCl_3_): *δ* (ppm) = 2.22 (s, 3H, C*H*_3_), 3.82 (s, 3H, OC*H*_3_), 3.90 (s, 3H, OC*H*_3_), 4.18 (s, 2H, SC*H*_2_), 7.00 (d, *J* = 7.91 Hz, 2H, Ar*H*), 7.19 (d, *J* = 8.19 Hz, 2H, Ar*H*), 7.56 (s, 1H, Ar*H*), 7.69 (s, 1H, Ar*H*), 8.05 (s, 1H, Ar*H*). ^13^C-NMR (100 MHz, CDCl_3_): *δ* (ppm) = 21.05 (*C*H_3_), 29.51 (S*C*H_2_), 56.88 (O*C*H_3_), 57.32 (O*C*H_3_), 109.99 (*C*_Ar_), 110.37 (*C*_Ar_), 124.17 (*C*_Ar_), 128.14 (*C*_Ar_), 129.20 (*C*_Ar_), 129.81 (*C*_Ar_), 130.70 (*C*_Ar_), 131.45 (*C*_Ar_), 136.94 (CH_3_*C*_Ar_), 137.97 (*C*_Ar_), 143.02 (*C*OCH_3_), 147.96 (*C*OCH_3_). MS (EI, 70 eV): *m*/*z* = 386.39, [M^+^]. Elemental analysis, calcd (%) for C_18_H_18_N_4_O_4_S: *C* 55.95, *H* 4.70, *N* 14.50, *S* 8.30; found *C* 56.07, *H* 4.40, *N* 14.35, *S* 8.44.

##### 2.1.2.3 1-(4-(Octyloxy)phenyl)-4-((*p*-tolylthio)methyl)-1*H*-1,2,3-triazole (12)

The title compound as a pale brown solid (94%). TLC (petroleum ether : ethyl acetate 8/2) *R*_f_ = 0.36. ^1^H-NMR (400 MHz, CDCl_3_): *δ* (ppm) = 0.80 (t, *J* = 6.78 Hz, 3H, C*H*_3_), 1.21–1.26 (m, 8H, C*H*_2_), 1.34–1.39 (m, 2H, C*H*_2_), 1.67–1.74 (m, 2H, C*H*_2_), 2.21 (s, 3H, C*H*_3_), 3.89 (t, *J* = 6.57 Hz, 2H, OC*H*_2_), 4.16 (S, 2H, SC*H*_2_), 6.86 (d, *J* = 8.92 Hz, 2H, Ar*H*), 6.99 (d, *J* = 7.76 Hz, 2H, Ar*H*), 7.17 (d, *J* = 7.97 Hz, 2H, Ar*H*), 7.43 (d, *J* = 8.91 Hz, 2H, Ar*H*), 7.60 (s, 1H, ArH). ^13^C-NMR (100 MHz, CDCl_3_): *δ* (ppm) = 14.12 (*C*H_3_), 21.04 (*C*H_3_), 22.66 (*C*H_2_), 26.01 (*C*H_2_), 29.16 (*C*H_2_), 29.23 (*C*H_2_), 29.35 (*C*H_2_), 29.50 (S*C*H_2_), 31.81 (*C*H_2_), 68.47 (O*C*H_2_), 115.25 (*C*_Ar_), 119.95 (*C*_Ar_), 122.05 (*C*_Ar_), 128.15 (*C*_Ar_), 129.83 (*C*_Ar_), 130.39 (*C*_Ar_), 131.68 (*C*_Ar_), 134.08 (*C*_Ar_), 136.79 (CH_3_*C*_Ar_), 159.38 (*C*OCH_2_). MS (EI, 70 eV): *m*/*z* = 410.57, [M^+^]. Elemental analysis, calcd. (%) for C_24_H_31_N_3_OS: *C* 70.38, *H* 7.63, *N* 10.26, *S* 7.83; found *C* 70.43, *H* 7.47, *N* 10.38, *S* 7.69.

##### 2.1.2.4 1-(4-(Octadecyloxy)phenyl)-4-((*p*-tolylthio)methyl)-1*H*-1,2,3-triazole (13)

The title compound as a beige solid (93%). TLC (petroleum ether : ethyl acetate 8/2) *R*_f_ = 0.35. ^1^H-NMR (400 MHz, CDCl_3_): *δ* (ppm) = 0.80 (t, *J* = 6.77 Hz, 3H, C*H*_3_), 1.18–1.38 (m, 30H, C*H*_2_), 1.68–1.75 (m, 2H, C*H*_2_), 2.22 (s, 3H, C*H*_3_), 3.90 (t, *J* = 6.69 Hz, 2H, OC*H*_2_), 4.18 (S, 2H, SC*H*_2_), 6.88 (d, *J* = 8.77 Hz, 2H, Ar*H*), 7.00 (d, *J* = 8.03 Hz, 2H, Ar*H*), 7.19 (d, *J* = 7.96 Hz, 2H, Ar*H*), 7.44 (d, *J* = 8.76 Hz, 2H, Ar*H*), 7.62 (s, 1H, ArH). ^13^C-NMR (100 MHz, CDCl_3_): *δ* (ppm) = 14.14 (*C*H_3_), 21.04 (*C*H_3_), 22.71 (*C*H_2_), 26.02 (*C*H_2_), 29.17 (*C*H_2_), 29.38 (*C*H_2_), 29.40 (*C*H_2_), 29.59 (S*C*H_2_), 29.62 (*C*H_2_), 29.64 (*C*H_2_), 29.68 (*C*H_2_), 29.72 (*C*H_2_), 31.94 (*C*H_2_), 68.49 (O*C*H_2_), 115.27 (*C*_Ar_), 122.09 (*C*_Ar_), 124.17 (*C*_Ar_), 128.53 (*C*_Ar_), 129.23 (*C*_Ar_), 129.84 (*C*_Ar_), 130.43 (*C*_Ar_), 131.61 (*C*_Ar_), 136.83 (CH_3_*C*_Ar_), 159.44 (*C*OCH_2_). MS (EI, 70 eV): *m*/*z* = 550.89, [M^+^+H]. Elemental analysis, calcd (%) for C_34_H_51_N_3_OS: *C* 74.27, *H* 9.35, *N* 7.64, *S* 5.83; found *C* 74.36, *H* 9.24, *N* 7.78, *S* 5.67.

##### 2.1.2.5 1-(2-(Octadecyloxy)phenyl)-4-((*p*-tolylthio)methyl)-1*H*-1,2,3-triazole (14)

The title compound as a beige solid (90%). TLC (petroleum ether : ethyl acetate 8/2) *R*_f_ = 0.34. ^1^H-NMR (400 MHz, CDCl_3_): *δ* (ppm) = 0.90 (t, *J* = 6.79 Hz, 3H, C*H*_3_), 1.28–1.37 (m, 30H, C*H*_2_), 1.70–1.73 (m, 2H, C*H*_2_), 2.31 (S, 3H, C*H*_3_), 4.01 (t, *J* = 6.44 Hz, 2H, OC*H*_2_), 4.30 (S, 2H, SC*H*_2_), 7.04–7.11 (m, 3H, Ar*H*), 7.28 (d, *J* = 8.17 Hz, 2H, Ar*H*), 7.36–7.40 (m, 2H, Ar*H*), 7.78 (d, *J* = 8.81 Hz, 1H, Ar*H*), 8.02 (s, 1H, Ar*H*). ^13^C-NMR (100 MHz, CDCl_3_): *δ* (ppm) = 14.14 (*C*H_3_), 21.03 (*C*H_3_), 22.71 (*C*H_2_), 26.04 (*C*H_2_), 28.98 (*C*H_2_), 29.32 (*C*H_2_), 29.39 (*C*H_2_), 29.51 (S*C*H_2_), 29.61 (*C*H_2_), 29.65 (*C*H_2_), 29.69 (*C*H_2_), 29.70 (*C*H_2_), 29.73 (*C*H_2_), 31.93 (*C*H_2_), 69.07 (O*C*H_2_), 112.85 (*C*_Ar_), 113.19 (*C*_Ar_), 121.02 (*C*_Ar_), 125.21 (*C*_Ar_), 126.49 (*C*_Ar_), 129.20 (*C*_Ar_), 129.75 (*C*_Ar_), 129.86 (*C*_Ar_), 129.99 (C_Ar_), 132.08 (C_Ar_), 136.44 (CH_3_*C*_Ar_), 150.44 (*C*OCH_2_). MS (EI, 70 eV): *m*/*z* = 549.86, [M^+^]. Elemental analysis, calcd (%) for C_34_H_51_N_3_OS: *C* 74.27, *H* 9.35, *N* 7.64, *S* 5.83; found *C* 74.15, *H* 9.43, *N* 7.49, *S* 6.02.

##### 2.1.2.6 Dioctyl 5-(4-((*p*-tolylthio)methyl)-1*H*-1,2,3-triazol-1-yl)isophthalate (15)

The title compound as a pale brown solid (85%). TLC (petroleum ether : ethyl acetate 8/2) *R*_f_ = 0.32. ^1^H-NMR (400 MHz, CDCl_3_): *δ* (ppm) = 0.80 (t, *J* = 6.72 Hz, 6H, C*H*_3_), 1.20–1.26 (m, 20H, C*H*_2_), 1.68–1.75 (m, 4H, C*H*_2_), 2.22 (s, 3H, C*H*_3_), 4.19 (s, 2H, SC*H*_2_), 4.30 (t, *J* = 6.78 Hz, 4H, CO_2_C*H*_2_), 7.00 (d, *J* = 7.90 Hz, 2H, Ar*H*), 7.18 (d, *J* = 8.06 Hz, 2H, Ar*H*), 7.79 (s, 1H, Ar*H*), 8.43 (s, 2H, Ar*H*), 8.61 (s, 1H, Ar*H*). ^13^C-NMR (100 MHz, CDCl_3_): *δ* (ppm) = 14.08 (*C*H_3_), 21.02 (*C*H_3_), 22.63 (*C*H_2_), 25.95 (*C*H_2_), 28.64 (*C*H_2_), 29.17 (*C*H_2_), 29.22 (*C*H_2_), 29.46 (S*C*H_2_), 31.78 (*C*H_2_), 66.15 (O*C*H_2_), 120.28 (*C*_Ar_), 124.90 (*C*_Ar_), 128.48 (*C*_Ar_), 129.20 (*C*_Ar_), 129.89 (*C*_Ar_), 130.21 (*C*_Ar_), 130.55 (*C*_Ar_), 131.32 (*C*_Ar_), 132.79 (*C*_Ar_), 137.05 (CH_3_*C*_Ar_), 164.62 (*C*O_2_). MS (EI, 70 eV): *m*/*z* = 593.84, [M^+^]. Elemental analysis, calcd. (%) for C_34_H_47_N_3_O_4_S: *C* 68.77, *H* 7.98, *N* 7.08, *S* 5.40; found *C* 68.89, *H* 7.79, *N* 7.21, *S* 5.28.

##### 2.1.2.7 (2*S*,3*S*,4*R*,5*S*)-2-(4-((*P*-tolylthio)methyl)-1*H*-1,2,3-triazol-1-yl)tetrahydro-2*H*-pyran-3,4,5-triyl triacetate (16)

The title compound was separated and purified by column chromatography (petroleum ether/ethyl acetate 8/2) as a dark brown solid (75%). TLC (petroleum ether/ethyl acetate 8/2) *R*_f_ = 0.30. ^1^H NMR (400 MHz, CDCl_3_): *δ* (ppm) = 1.96, 1.97, 1.98 (sss, 9H, 3COC*H*_3_), 2.25 (s, 3H, C*H*_3_), 3.28–3.33 (m, 1H, C*H*), 3.39 (s, 2H, SC*H*_2_), 4.03–4.07 (m, 1H, C*H*), 4.32 (d, ^2^*J* = 6.78 Hz, 1H, C*H*), 4.81–4.90 (m, 2H, 2C*H*), 5.09 (t, ^3^*J* = 8.57 Hz, 1H, C*H*), 7.05 (d, ^2^*J* = 7.94 Hz, 2H, Ar*H*), 7.28 (d, ^2^*J* = 8.13 Hz, 2H, Ar*H*), 7.50 (s, 1H, Ar*H*_triazole_). ^13^C NMR (75 MHz, CDCl_3_): *δ* (ppm) = 20.67 (CO*C*H_3_), 20.70 (CO*C*H_3_), 21.05 (CO*C*H_3_), 23.21 (*C*H_3_), 29.44 (S*C*H_2_), 61.95 (*C*H), 68.92 (*C*H), 70.74 (*C*H_2_), 71.47 (*C*H), 101.54 (N-*C*_suger_), 128.51 (*C*_Ar_), 129.13 (*C*_Ar_), 129.74 (*C*_Ar_), 130.51 (*C*_Ar_), 131.01 (*C*_Ar_), 137.29 (CH_3_*C*_Ar_), 169.49, 169.85, 170.08 (3*C* = O). Elemental analysis, Calc. (%) for C_21_H_25_N_3_O_7_S: *C* 54.42, *H* 5.44, *N* 9.07, *S* 6.92; found: *C* 54.27, *H* 5.63, *N* 8.95, *S* 7.13.

##### 2.1.2.8 (2*S*,3*R*,4*R*,5*S*,6*S*)-2-(Acetoxymethyl)-6-(4-((*p*-tolylthio)methyl)-1*H*-1,2,3-triazol-1-yl)tetrahydro-2*H*-pyran-3,4,5-triyl triacetate (17)

The title compound was separated and purified by column chromatography (petroleum ether/ethyl acetate 8/2) as a pale brown solid (71%). TLC (petroleum ether/ethyl acetate 8/2) *R*_f_ = 0.28. ^1^H NMR (400 MHz, CDCl_3_): *δ* (ppm) = 1.79 (s, 3H, COC*H*_3_), 1.96 (s, 3H, COC*H*_3_), 1.99 (s, 3H, COC*H*_3_), 2.17 (s, 3H, COC*H*_3_), 2.27 (s, 3H, C*H*_3_), 4.07–4.12 (m, 1H, C*H*), 4.14 (s, 2H, SC*H*_2_), 4.17–4.22 (m, 1H, C*H*), 4.23–4.25 (m, 1H, C*H*), 5.23–5.26 (m, 1H, C*H*), 5.47–5.51 (m, 2H, 2C*H*), 5.80 (d, ^2^*J* = 9.29 Hz, 1H, C*H*), 7.04 (d, ^2^*J* = 7.56 Hz, 2H, Ar*H*), 7.20 (d, ^2^*J* = 7.33 Hz, 2H, Ar*H*), 7.65 (s, 1H, Ar*H*_triazole_). ^13^C NMR (75 MHz, CDCl_3_): *δ* (ppm) = 20.13 (COCH_3_), 20.45 (COCH_3_), 20.60 (COCH_3_), 20.99 (COCH_3_), 29.95 (*C*H_3_), 31.18 (S*C*H_2_), 61.21 (CH_2_), 66.96 (CH), 67.81 (CH), 70.79 (CH), 73.88 (CH), 86.08 (N-*C*_suger_), 120.60 (C_Ar_), 128.49 (C_Ar_), 129.74 (C_Ar_), 130.50 (*C*_Ar_), 131.56 (C_Ar_), 136.74 (CH_3_*C*_Ar_), 168.90, 169.78, 169.97, 170.30 (4*C* = O). Elemental analysis, calc. (%) for C_24_H_29_N_3_O_9_S: *C* 53.82, *H* 5.46, *N* 7.85, *S* 5.99; found: *C* 53.64, *H* 5.58, *N* 7.68, *S* 6.13.

### 2.2 Biology

#### 2.2.1 Materials

For the purposes of this research, RPMI 1640 medium was sourced from Sigma Chemical Company, located in St. Louis, Missouri, USA. The fetal bovine serum (FBS) and fetal calf serum (FCS) were obtained from Gibco, a prominent supplier in the United Kingdom. Dimethyl sulfoxide (DMSO) and methanol were utilized in their HPLC grade forms, and all other reagents and chemicals were of analytical reagent grade quality.

#### 2.2.2 Cell culture

The cell lines utilized in this study were HepG-2, associated with human liver carcinoma, HCT116, a model for human colorectal carcinoma, and MCF-7, linked to human breast adenocarcinoma. These cell lines were obtained from the American Type Culture Collection in Rockville, MD, USA. They were cultured in RPMI-1640 medium supplemented with 10% heat-inactivated FBS, and 100 U per ml of both penicillin and streptomycin. Culturing took place at 37 °C in a controlled atmosphere with 5% CO_2_. Each experiment was repeated three times, and all values represented as means ± standard deviation (SD).

#### 2.2.3 Lactate dehydrogenase (LDH) assay

To evaluate the cytotoxic effects of the synthesized compounds on cellular membrane integrity, an LDH release assay was conducted using HepG2, MCF-7, and HCT-116 human cancer cell lines, as well as the non-cancerous BJ-1 fibroblast cell line.^31–34^ Cells were cultured in 24-well plates at a density of 2 × 10^5^ cells per well in a 500 µL medium and incubated for 18 hours to allow proper attachment. Following this, the cells were exposed to varying concentrations of the test compounds or Doxorubicin® as a positive control for 48 hours. After the incubation period, 40 µL of the culture supernatant was collected to measure extracellular LDH, while 40 µL of 6% Triton X-100 was added to the remaining cells to release total intracellular LDH. LDH activity was then assessed by adding 100 µL of a pyruvic acid solution in potassium phosphate buffer (pH 7.5), followed by 100 µL of β-NADH solution in the same buffer. Absorbance at 340 nm was recorded kinetically over one minute using a microplate reader. The percentage of LDH released was determined by dividing the amount in the supernatant by the total LDH after cell lysis, reflecting the degree of cytotoxicity induced by each compound.

#### 2.2.4 Statistical analysis

Each experiment was independently repeated three times (*n* = 3), with data presented as the mean ± standard deviation (SD). The half-maximal inhibitory concentration (IC_50_) for each compound was determined using probit analysis. To assess the statistical significance of differences in cytotoxicity between the synthesized derivatives and the reference drug doxorubicin, the mean IC_50_ values for each cell line were compared using one-way analysis of variance (ANOVA) followed by Dunnett's post hoc test. A *p*-value of less than 0.05 was considered statistically significant. All statistical analyses were performed using SPSS software (SPSS Inc., Chicago, IL, USA).

### 2.3 Docking studies

The X-ray defined protein structure of epidermal growth factor receptor (EGFR) kinase domain was obtained from https://www.rcsb.org (the protein data bank website) PDB ID: 4wkq. The co-crystalized ligand (gefitinib) and water molecules were eliminated then the prepared protein was saved as PDB file by BioviaDiscoverstudio2021 for the following steps. CB-DOCK webserver (http://clab.labshare.cn/cb-dock/php/) was accessed and used for active site detection and molecular docking following its default protocol.^35^ Compounds 10–17 were docked against 4WKQ at grid box dimensions *x*: 17.77, *y*: 191.57, *z*: 18.13.

## 3 Results and discussion

### 3.1 Chemistry

**Fig. 2:**
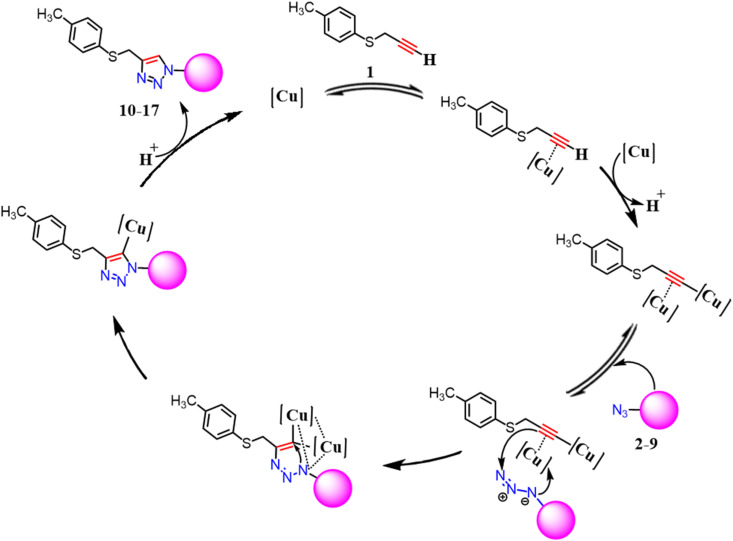
Proposed mechanism for the formation 4-((*p*-tolylthio)methyl)-1*H*-1,2,3-triazole derivatives (10–17).

**Scheme 1:**
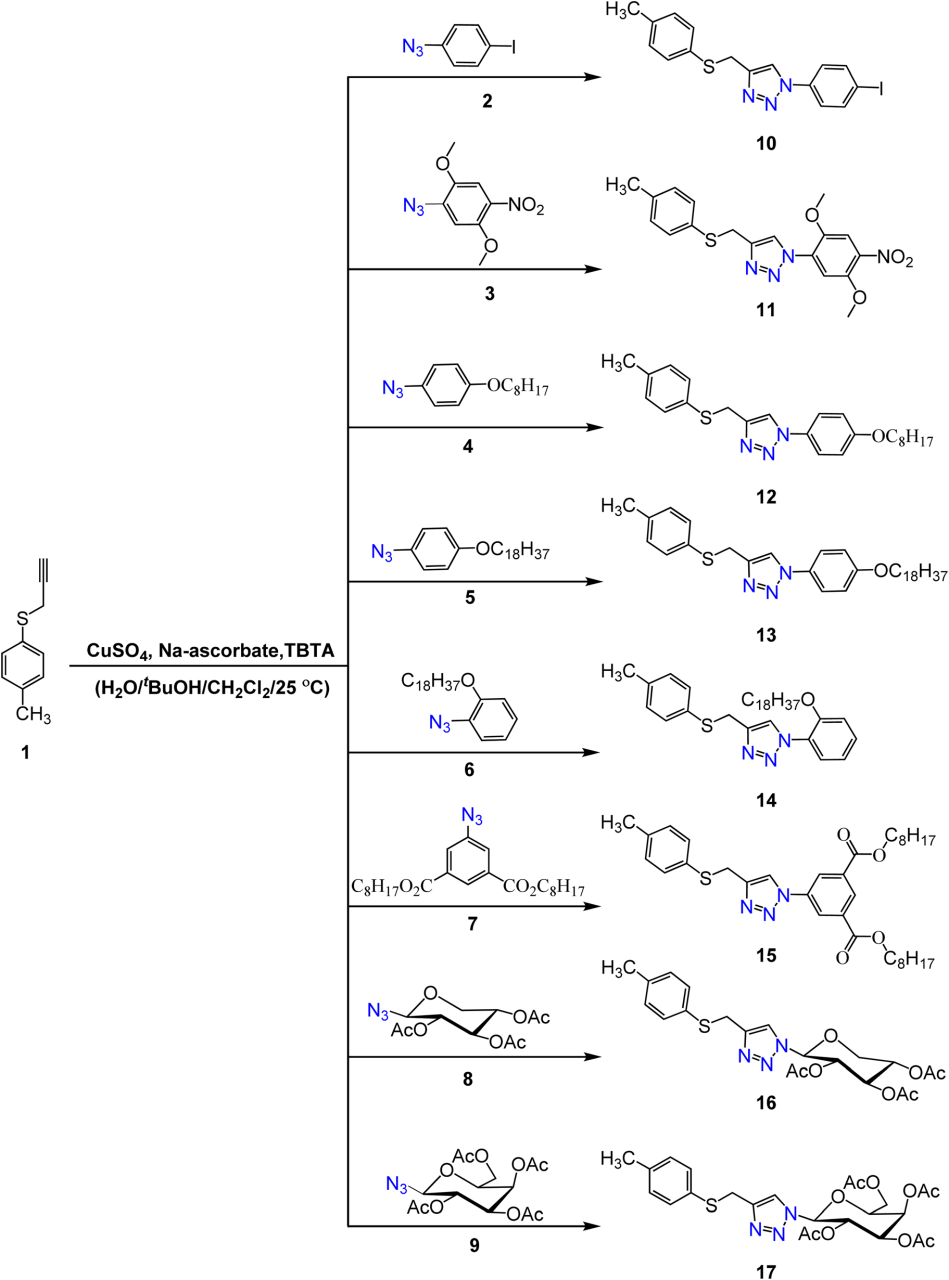
The synthetic route for the formation of 4-((*p*-tolylthio)methyl)-1*H*-1,2,3-triazole derivatives (10–17).

A new series of 4-((*p*-tolylthio)methyl)-1*H*-1,2,3-triazole hybrids 10–17 was synthesized through the copper-catalyzed azide–alkyne cycloaddition (CuAAC) reactions between prop-2-yn-1-yl(*p*-tolyl)sulfane 1 (ref. 36) and aromatic azides 2–9.^31,32,37,38^ This reaction takes place in the presence of CuSO_4_ sodium ascorbate as a reducing agent, and tri(benzyl-triazolyl methyl)amine (TBTA) as a stabilizing ligand for Cu(i). The structures of the obtained products were characterized based on their elemental analysis and different spectroscopic techniques. Generally, in the ^1^H NMR spectra of the compounds 10–17 revealed the absence of the proton characterized for (C≡C*H*) and the presence of singles at *δ* 3.39–4.30 and 7.50–8.02 ppm attributed to SCH_2_ and the triazole-H groups, respectively. In the ^13^C NMR spectra of these compounds, S-*C*H_2_ junction groups appeared at *δ* 29.44–29.59 ppm and the aromatic carbons of triazole rings were observed at *δ* 119.95–122.09 ppm and at *δ* 131.45–132.08 ppm. The remaining protons and carbons of the pyridinyl and substituted phenyl groups in both ^1^H/^13^C NMR spectra resonated at their usual chemical shifts. Finally, the mass spectra of the obtained compounds showed M^+^ confirmed their chemical structures (*cf.* Experimental). The proposed mechanism for the formation of compounds 10–17 is shown in Fig. 2.^39^ For example, the ^1^H NMR spectrum of compound 10 exhibited singlet signal at *δ* 2.21 ppm characteristic for terminal C*H*_3_ group and the methylene (SC*H*_2_) that links oxadiazole and triazole appeared downfield at *δ* 4.16 ppm. Furthermore, the triazole (C*H*) signal was observed at δ 7.66 ppm. The remaining protons of the (*p*-tolyl)sulfane and 4-iodo-benzene groups resonated at their usual chemical shifts. The ^13^C NMR spectrum of compound 10 revealed signals at *δ* 29.44 due to carbon of –S*C*H_2_ linker and signal at *δ* 21.08 ppm accountable for the C*H*_3_ group. Also, the MS gave the molecular ion peaks at *m*/*z* = 407.32, [M^+^], supporting the proposed structure of compound 10 (Scheme 1).

### 3.2 *In vitro* antiproliferative activity

**Fig. 3:**
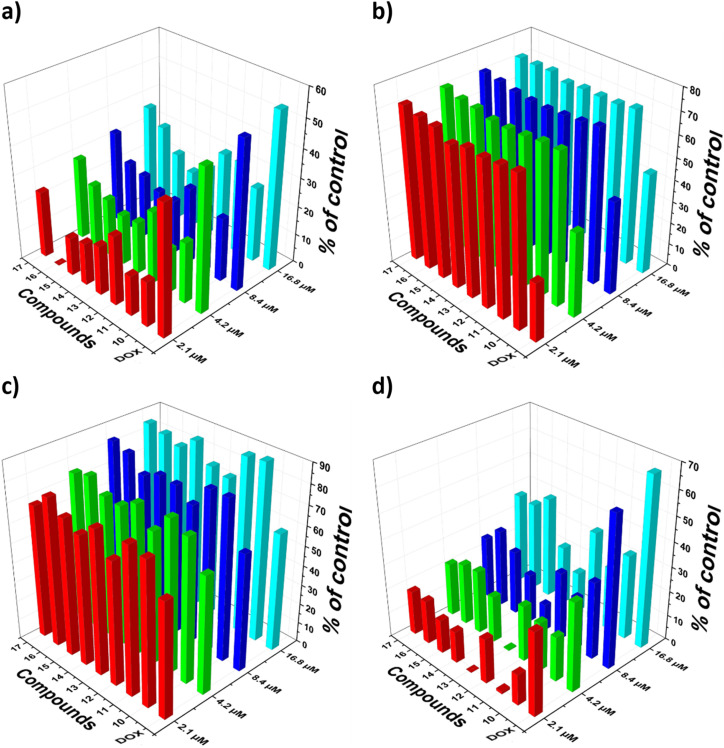
Designed dose-dependent antiproliferative data of the new 1,2,3-triazoles on the different human cancer types (a) HCT-116, (b) HepG-2, (c) MCF-7, and (d) BJ-1, according to the LDH assay after 48 h of exposure.

*In vitro* assessments were conducted on eight synthesized compounds to evaluate their cytotoxic activity against three human cancer cell lines: HCT-116 (colorectal carcinoma), HepG2 (hepatocellular carcinoma), and MCF-7 (breast adenocarcinoma), as well as a normal human fibroblast cell line (BJ-1), using the lactate dehydrogenase (LDH) release assay. The cell death percentages were calculated compared to the untreated control, and the results were justified with those of the standard chemotherapeutic agent, doxorubicin. The compounds evaluated demonstrated a dose-dependent cytotoxic response across the three cancer cell lines, as shown in Fig. 3. In the case of HCT-116 cells (Fig. 3a and Table 1), the compounds exhibited moderate levels of cytotoxicity relative to doxorubicin. In contrast, a stronger cytotoxic response was observed in HepG2 and MCF-7 cells, with several compounds surpassing doxorubicin (Fig. 3b, c and Table 1). Crucially, a low level of cytotoxicity was recorded against the BJ-1 normal fibroblast cell line, indicating a favorable selectivity for cancer cells compared to healthy cells (Fig. 3 and Table 1).

**Table 1:** The anticancer IC_50_ of compounds 10–17 derivatives against the four cell lines according to the LDH assay

Compound no.	IC_50_ (µM) ± SD				*p*-Value *vs.* Dox		
	HCT-116	HepG-2	MCF-7	BJ-1	HCT-116	HepG-2	MCF-7
10	32.1 ± 3.1	1.5 ± 0.1	1.5 ± 0.1	24.6 ± 2.2	<0.001	**<0.001**	0.12
11	26.0 ± 2.9	1.6 ± 0.2	1.4 ± 0.1	32.7 ± 3.3	<0.001	**<0.001**	**0.03**
12	25.9 ± 2.1	1.6 ± 0.2	1.7 ± 0.2	22.8 ± 2.1	<0.001	**<0.001**	0.65
13	22.0 ± 2.3	1.6 ± 0.2	1.4 ± 0.1	51.0 ± 4.1	<0.001	**<0.001**	**0.02**
14	41.6 ± 3.4	1.6 ± 0.2	1.6 ± 0.2	35.0 ± 3.2	<0.001	**<0.001**	0.42
15	35.0 ± 3.3	1.5 ± 0.1	1.5 ± 0.1	20.4 ± 2.5	<0.001	**<0.001**	0.10
16	26.9 ± 2.1	1.5 ± 0.1	1.4 ± 0.1	23.3 ± 2.1	<0.001	**<0.001**	**0.04**
17	23.4 ± 1.9	1.5 ± 0.1	1.5 ± 0.2	22.8 ± 2.1	<0.001	**<0.001**	0.08
Doxorubicin	8.1 ± 0.7	3.8 ± 0.3	1.8 ± 0.2	6.9 ± 0.5	—	—	—

While the LDH assay effectively identified compounds with potent and selective cytotoxicity, it measures general membrane integrity loss and does not specify the mode of cell death. The pronounced activity of compounds 13 and 17 against HepG-2 and MCF-7 cells warrants further investigation using complementary assays (*e.g.*, for apoptosis detection) to delineate the precise mechanistic pathways responsible for the observed cell death. Statistical comparison to doxorubicin using one-way ANOVA with Dunnett's post hoc test revealed that the superior activity of most compounds against HepG-2 cells was highly significant (*p* < 0.001, Table 1). Notably, compounds 11, 13, and 16 also demonstrated statistically significant greater potency than doxorubicin against MCF-7 cells (*p* < 0.05). As anticipated, all synthesized compounds were significantly less potent than doxorubicin against HCT-116 cells (*p* < 0.001).

### 3.3 Molecular docking studies

**Fig. 4:**
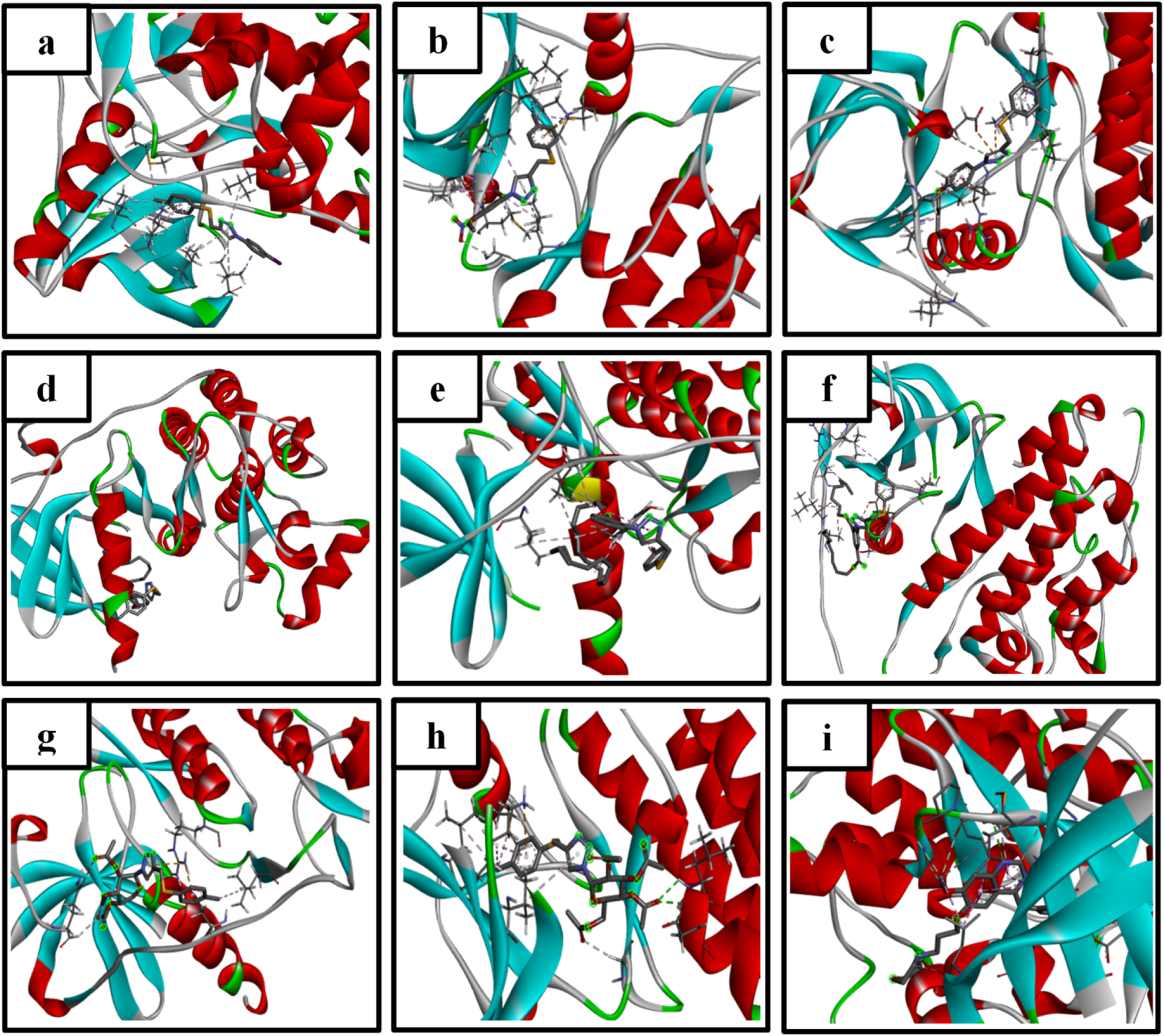
3D representation of compounds (a) 10, (b) 11, (c) 12, (d) 13, (e) 14, (f) 15, (g) 16, (h) 17, and (i) co-crystalized inhibitor interactions with EGFR kinase.

**Fig. 5:**
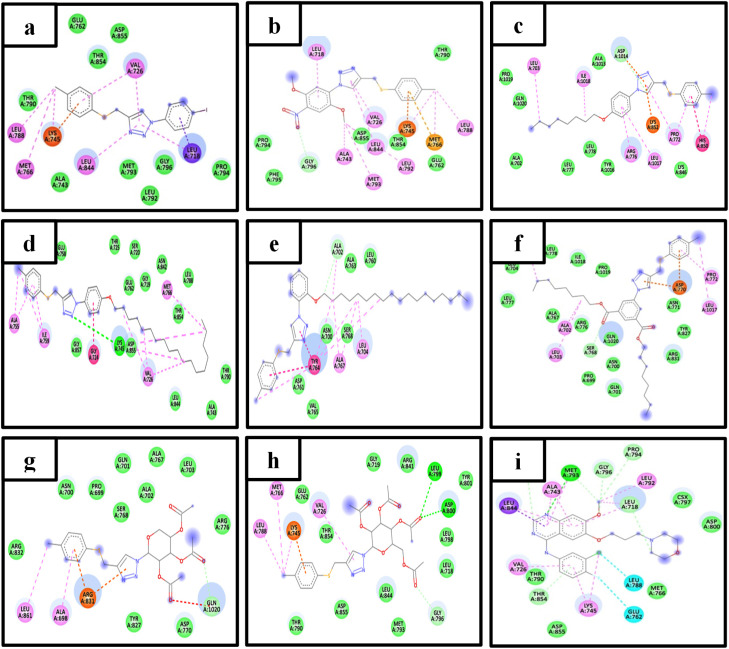
2D representation of compounds (a) 10, (b) 11, (c) 12, (d) 13, (e) 14, (f) 15, (g) 16, (h) 17, and (i) co-crystalized inhibitor interactions with EGFR kinase.

Epidermal growth factor receptors (EGFRs) are a class of receptor tyrosine kinase. The inhibition of EGFR tyrosine kinase activity is a hopeful therapeutic approach for cancer treatment.^40^ In the current study, the synthesized compounds were docked against the EGFR kinase and yielded some of interactions (summarized in Table 2) and shown in Fig. 4 and 5. From which, it could be noticed that most of the compounds could extend within the active site of the kinase domain of the EGFR making similar interactions to the Gefitinib, (the FDA approved EGFR inhibitor, anticancer drug and the co-crystalized ligand of 4WKQ) like in the interactions with residues leu718, leu844, ala743, val726, lys745 and leu788 that varied in strength and nature (Pi cation, Pi alkyl, Pi anion, Pi sulfur, van der Waals and C–H bonds). Among the new compounds, only compounds 13 and 17 could form H bonds (lys745, leu799 & asp800) that greatly add to the stability of the tested compounds in the EGFR pocket that could potentially enhance stability within the EGFR pocket, suggesting a possible basis for inhibitory activity. The docking results suggest that compounds 13 and 17 may inhibit EGFR kinase activity, providing a testable hypothesis for their mechanism of action that should be pursued in future studies.

**Table 2:** Docking results of synthesized compounds 10–17 & the co-crystalized inhibitor with EGFR kinase (Pdb id: 4wkq)

Compound	Binding energy (kcal mol^−1^)	H-bonds		Residual interactions
		Number	Residues	
10	−6.1	(0)	—	Leu788- lys745- met766 -leu844 val726- leu718- lys745
11	−7.1	(0)	—	Leu718- ala743-asp 855 -leu844 val726- lys745- met766 -leu788- met793 - leu792-ala743- gly796
12	−6.4	(0)	—	Leu703- Ile1018- lys852- arg 776 - Leu1017-Pro772- HiS850- asp1014
13	−5.6	(1)	Lys745	Ala755-Ile759 gly724- val726-met766
14	−5.1	(0)	—	ALa702- leu 704- ala767- tyr764
15	−5.9	(0)	—	Arg70- ala702- Leu703- asp770 -Leu1017- pro 772 - ser768
16	−6.6	(0)	—	Leu 861- ala 698- arg 831- gln 1020
17	−6.7	(2)	Leu799 – Asp800	Lys745- Leu788- met766 - val726- gly796
Co-crystalized inhibitor	−8.3	(1)	Met793	Leu792- leu718- leu844 - ala743- val726- lys745- leu788- glu762- gly796 -gln791-

### 3.4 Molinspiration-based molecular descriptors and drug-likeness evaluation

The provided Table 1 summarizes key molecular descriptors computed *via* Molinspiration for a series of compounds (10–17) Table 3. Analysis reveals varying degrees of compliance with the Lipinski Rule of Five. Compounds 10, 11, and 16 show zero violations, suggesting favorable drug-like properties. In contrast, compounds 12 through 15 and 17 exhibit one or more violations, primarily due to high Log *P* values (exceeding 5) and, in some cases, elevated molecular weight (MW > 500). Notably, compounds 13, 14, and 15 each have two violations and significantly high molecular volumes and rotatable bond counts (*n* rotb = 22), which may indicate potential challenges with oral bioavailability and metabolic stability. The topological polar surface area (TPSA) varies widely, from 30.72 to 135.94 Å^2^, influencing predictions for membrane permeability. This dataset effectively illustrates how computational descriptors can profile and differentiate compounds based on their predicted absorption and drug-likeness.^41–43^

**Table 3:** Molinspiration molecular descriptors prediction

No.	MiLogp	TPSA	*n* atoms	MW	*n* ON	*n* OHNH	*n* viol	*n* rotb	Volume
10	4.93	30.72	21	407.28	3	0	0	4	279.40
11	4.20	95.01	27	386.43	8	0	0	7	329.83
12	7.36	39.95	29	409.60	4	0	1	12	398.56
13	9.69	39.95	39	549.87	4	0	2	22	566.58
14	9.72	39.95	39	549.87	4	0	2	22	566.58
15	9.49	83.33	42	593.83	7	0	2	22	579.69
16	2.57	118.87	32	463.51	10	0	0	10	399.84
17	2.37	135.94	36	519.58	11	0	2	12	452.21

### 3.5 Structure–activity relationship (SAR) analysis

The synthesized hybrids share a common pharmacophore consisting of a 1,4-disubstituted 1,2,3-triazole core linked to a (*p*-tolylthio)methyl moiety. The primary structural variation lies in the N1-substituent (*R*) on the triazole ring, which ranges from simple aryl groups to long alkyl chains and polar carbohydrate derivatives. Analysis of the cytotoxicity data (Table 1) and computed physicochemical properties (Molinspiration, Table 3) reveals preliminary trends linking structure to potency and selectivity.

#### 3.5.1 Influence of lipophilicity and long alkyl chains

A clear trend emerges when correlating antiproliferative activity with lipophilicity (expressed as MiLogP). Against the sensitive HepG-2 and MCF-7 cell lines, high lipophilicity appears favorable for potency. Compounds 13 and 14, featuring a long octadecyloxy (C_18_) chain (MiLogP ∼9.7), consistently exhibited potent activity (IC_50_ ≈ 1.6 µM). This suggests that enhanced membrane permeability or strong hydrophobic interactions within a target binding pocket contribute to their efficacy. Notably, compound 13 (with a *para*-octadecyloxy phenyl group) demonstrated the highest selectivity index against normal BJ-1 fibroblasts among the series (IC_50_ = 51.0 µM), implying that optimal hydrophobic bulk can improve cancer cell specificity. The shorter octyloxy analogue 12 (MiLogP 7.36) maintained good potency, indicating that a minimum alkyl chain length is sufficient for activity. However, the diester derivative 15, despite high lipophilicity (MiLogP 9.49), showed reduced selectivity, suggesting that the nature of the hydrophobic group (flexible chain *vs.* rigid aromatic diester) also impacts the therapeutic window.

#### 3.5.2 Role of aromatic substitution patterns

The electronic and steric nature of the aryl substituent modulates activity. The 4-iodophenyl derivative 10 (electron-deficient, moderately lipophilic) displayed potent activity against HepG-2/MCF-7 but lower selectivity against BJ-1 cells. In contrast, the 2,5-dimethoxy-4-nitrophenyl analogue 11 (incorporating both electron-donating and -withdrawing groups, lower LogP) showed excellent potency and statistically significant superiority over doxorubicin against MCF-7 cells. This indicates that polar, hydrogen-bond accepting groups (nitro, methoxy) can be beneficial for interaction with biological targets without compromising potency, potentially by balancing solubility.

#### 3.5.3 Impact of polar and bulky substituents

Introducing polar, bulky sugar moieties (compounds 16 and 17) significantly reduced calculated lipophilicity (MiLogP ∼2.5) while retaining potent cytotoxic activity. This demonstrates that high lipophilicity is not an absolute requirement for potency within this series. The retained activity of 16 and 17 likely stems from their ability to engage in extensive hydrogen bonding, as corroborated by the docking results for 17, which showed additional H-bonds with EGFR residues. The preserved activity of these polar derivatives against cancer cells, coupled with their low toxicity to BJ-1 cells, highlights the potential to modulate pharmacokinetic properties without losing efficacy.

#### 3.5.4 Selectivity for cancer *vs.* normal cells

A key observation is the general trend of low cytotoxicity against BJ-1 fibroblasts (IC_50_ > 20 µM) for all derivatives, compared to their sub-micromolar activity against HepG-2/MCF-7. This suggests inherent selectivity conferred by the core (*p*-tolylthio)methyl-triazole scaffold. The selectivity was most pronounced for compounds 13 (long alkyl chain) and 11 (polar nitro/methoxy groups), indicating that both extreme hydrophobicity and the introduction of polar functionalities can enhance the differential toxicity between cancerous and normal cells. This warrants further investigation into the uptake mechanisms or differential metabolic activation in specific cell types.

#### 3.5.5 Activity against HCT-116 cells

All compounds were significantly less active against the colorectal carcinoma HCT-116 line than against HepG-2/MCF-7. This cell line-specific resistance suggests that the mechanism of action or cellular uptake may depend on pathways differentially expressed in these cancer types. The uniform moderate activity across all structural variants indicates that the core pharmacophore alone is insufficient for potency against HCT-116, and future optimization for broad-spectrum activity may require distinct structural modifications.

## 4 Conclusions

In this study, a series of novel (*p-tolylthio)methyl-linked 1,2,3-triazole hybrids 10–17 were successfully synthesized *via* click chemistry. The compounds demonstrated potent and selective cytotoxicity *in vitro*, particularly against HepG2 and MCF-7 cancer cell lines, while exhibiting lower toxicity toward normal fibroblasts. Molecular docking analysis provided a plausible, yet hypothetical, mechanistic basis by suggesting favorable interactions with the EGFR kinase domain, with compounds 13 and 17 forming additional stabilizing hydrogen bonds. It is important to acknowledge that the biological evaluation relies on a single cytotoxicity assay (LDH), which does not specify the mode of cell death or confirm target engagement. Therefore, these findings identify (*p-tolylthio)methyl-triazole hybrids as promising scaffolds for anticancer drug discovery. Future work will focus on validating the mechanism of action through apoptosis assays and direct EGFR inhibition studies, followed by *in vivo* evaluation and further structural optimization.

## Author contributions

Tamer El Malah: conceptualization, methodology, formal analysis, writing – original draft, and writing – review & editing. Ahmed A. El-Rashedy: methodology, writing – review & editing of docking studies. Randa E. Abdel-Mageid: methodology, investigation. Aymn E. Rashad: conceptualization, writing – original draft, and writing – review & editing. Hanan A. Soliman: formal analysis and writing – original draft. Hanem M. Awad: methodology & anticancer studies review. Ahmed H. Shamroukh: conceptualization, writing – original draft, and writing – review & editing.

## Conflicts of interest

No potential conflict of interest was reported by the authors.

## Supplementary Material

RA-016-D5RA09528J-s001

## Data Availability

All data generated or analyzed during this study are included in this published article and its supplementary information (SI). Supplementary information is available. See DOI: https://doi.org/10.1039/d5ra09528j.
