# Supplementary material for: Triazole-(p-tolylthio)methyl hybrids via click chemistry: synthesis, molecular docking, and evaluation as promising anticancer candidates
Source: RSC Adv. 2026 Jan 20;16(5):4287–98. doi: 10.1039/d5ra09528j (PMC12818029; doi:10.1039/d5ra09528j)
Supplement: RA-016-D5RA09528J-s001 [file RA-016-D5RA09528J-s001.pdf]

Supplementary Information (SI) for RSC Advances.
This journal is © The Royal Society of Chemistry 2026

Triazole-(*p*-tolylthio)methyl Hybrids *via* Click Chemistry: Synthesis, Molecular Docking, and Evaluation as Promising Anticancer Candidates

Tamer El Malah[a] *, Ahmed A. El-Rashedy[b,c], Randa E. Abdel-Mageid[a], Aymn E. Rashad[a], Hanan A. Soliman[a], Hanem M. Awad[d] and Ahmed H. Shamroukh[a]

[a] *Photochemistry Department, Chemical Industries Research Institute, National Research Centre, 33 El Buhouth Street, P.O. Box 12622, Cairo, Egypt*
[b] *Department of Organic and Medicinal Chemistry, Faculty of Pharmacy, University of Sadat City, Menoufia 32897, Egypt*
[c] *Chemistry of Natural and Microbial Products Department, National Research Centre, Dokki, 12622 Cairo, Egypt*
[d] *Department of Tanning Materials and Leather Technology, National Research Centre, 33 El Buhouth Street, P.O. Box 12311, Cairo, Egypt.*

*Corresponding author: tmara_nrc3000@yahoo.com (Tamer El Malah)

Table of Contents

Figure S1. ^{1}H NMR spectrum of compound **10** (400 MHz, $CDCl_3$, 25 °C)…………………….. **3**

Figure S2. ^{13}C NMR spectrum of compound **10** (100 MHz, CDCl3, 25 °C)…………………….. **3**

Figure S3. ^{1}H NMR spectrum of compound **11** (400 MHz, $CDCl_3$, 25 °C)……………………… **4**

Figure S4. ^{13}C NMR spectrum of compound **11** (100 MHz, $CDCl_3$, 25 °C)……………………… **4**

Figure S5. ^{1}H NMR spectrum of compound **12** (100 MHz, $CDCl_3$, 25 °C)……………………….. **5**

Figure S6. ^{13}C NMR spectrum of compound **12** (400 MHz, $CDCl_3$, 25 °C)……………………….. **5**

Figure S7. ^{1}H NMR spectrum of compound **13** (400 MHz, $CDCl_3$, 25 °C)……………………….. **6**

Figure S8. ^{13}C NMR spectrum of compound **13** (100 MHz, $CDCl_3$, 25 °C)……………………….. **6**

Figure S9. ^{1}H NMR spectrum of compound **14** (400 MHz, $CDCl_3$, 25 °C)……………………….. **7**

Figure S10. ^{13}C NMR spectrum of compound **14** (100 MHz, $CDCl_3$, 25 °C)……………………… **7**

Figure S11. ^{1}H NMR spectrum of compound **15** (400 MHz, $CDCl_3$, 25 °C)……………………… **8**

Figure S12. ^{13}C NMR spectrum of compound **15** (100 MHz, $CDCl_3$, 25 °C)……………………… **8**

Figure S13. ^{1}H NMR spectrum of compound **16** (400 MHz, $CDCl_3$, 25 °C)…………………….. **9**

Figure S14. ^{13}C NMR spectrum of compound **16** (100 MHz, $CDCl_3$, 25 °C)…………………….. **9**

Figure S15. ^{1}H NMR spectrum of compound **17** (400 MHz, $CDCl_3$, 25 °C)…………………….. **10**

Figure S16. ^{13}C NMR spectrum of compound **17** (100 MHz, $CDCl_3$, 25 °C)……………………… **10**

Compounds 10

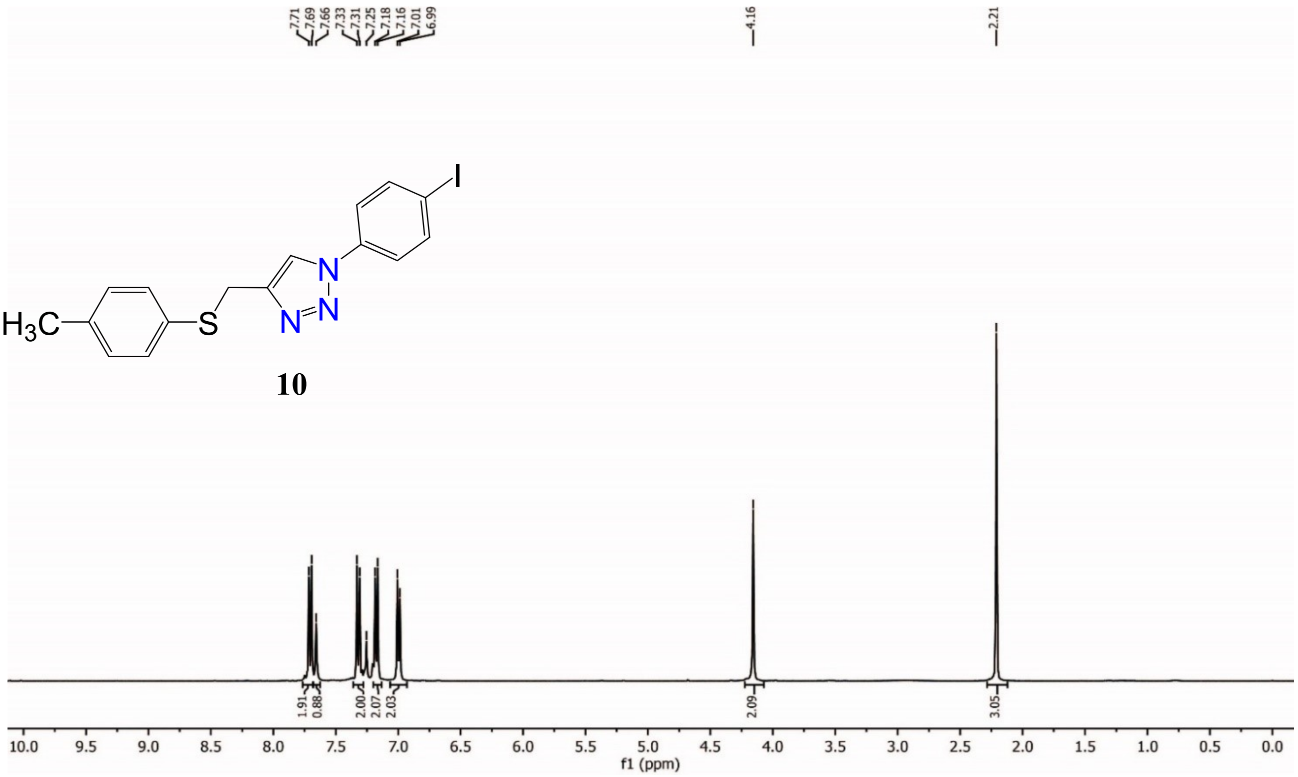

Figure S1. ^{1}H NMR spectrum of compound **10** (400 MHz, $CDCl_3$, 25 °C)……………………...

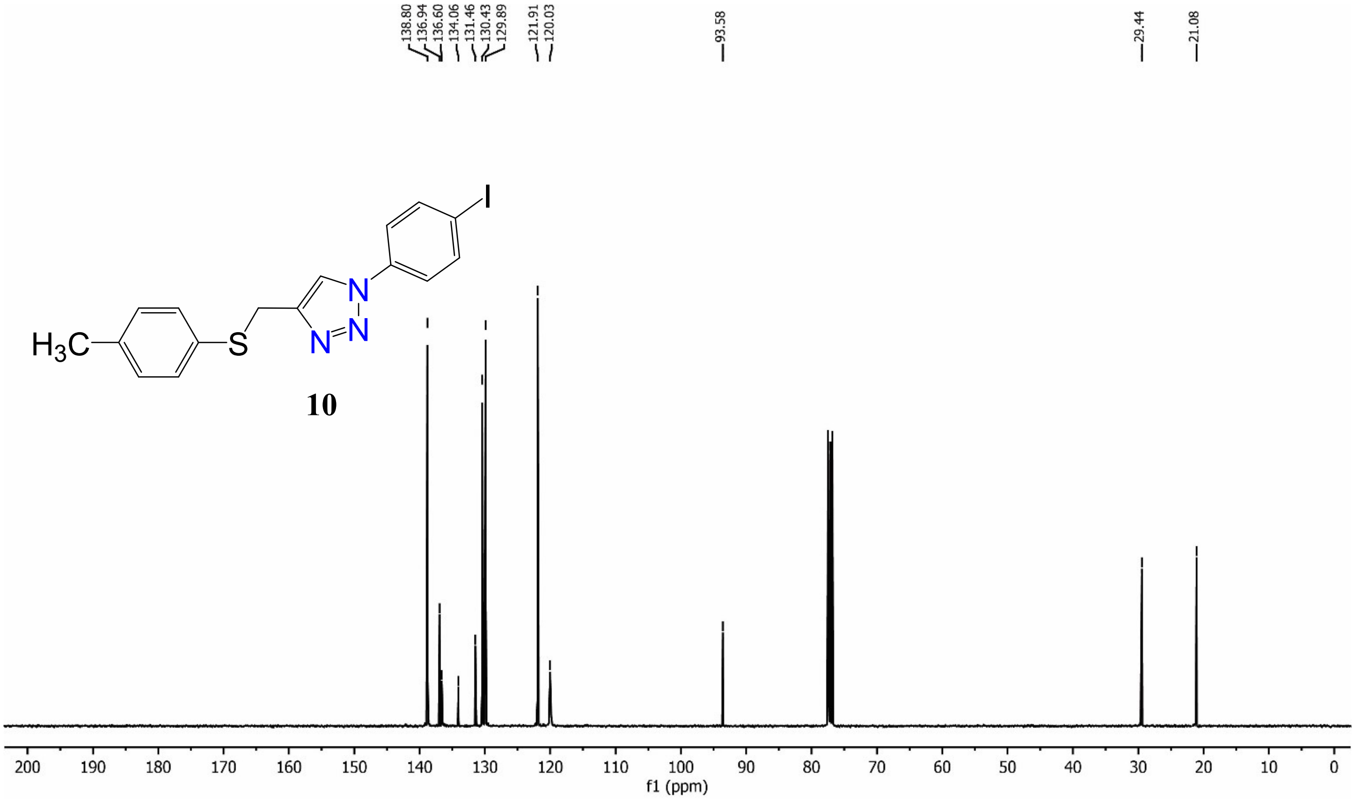

Figure S2. ^{13}C NMR spectrum of compound **10** (100 MHz, CDCl3, 25 °C)……………………..

Compounds 11

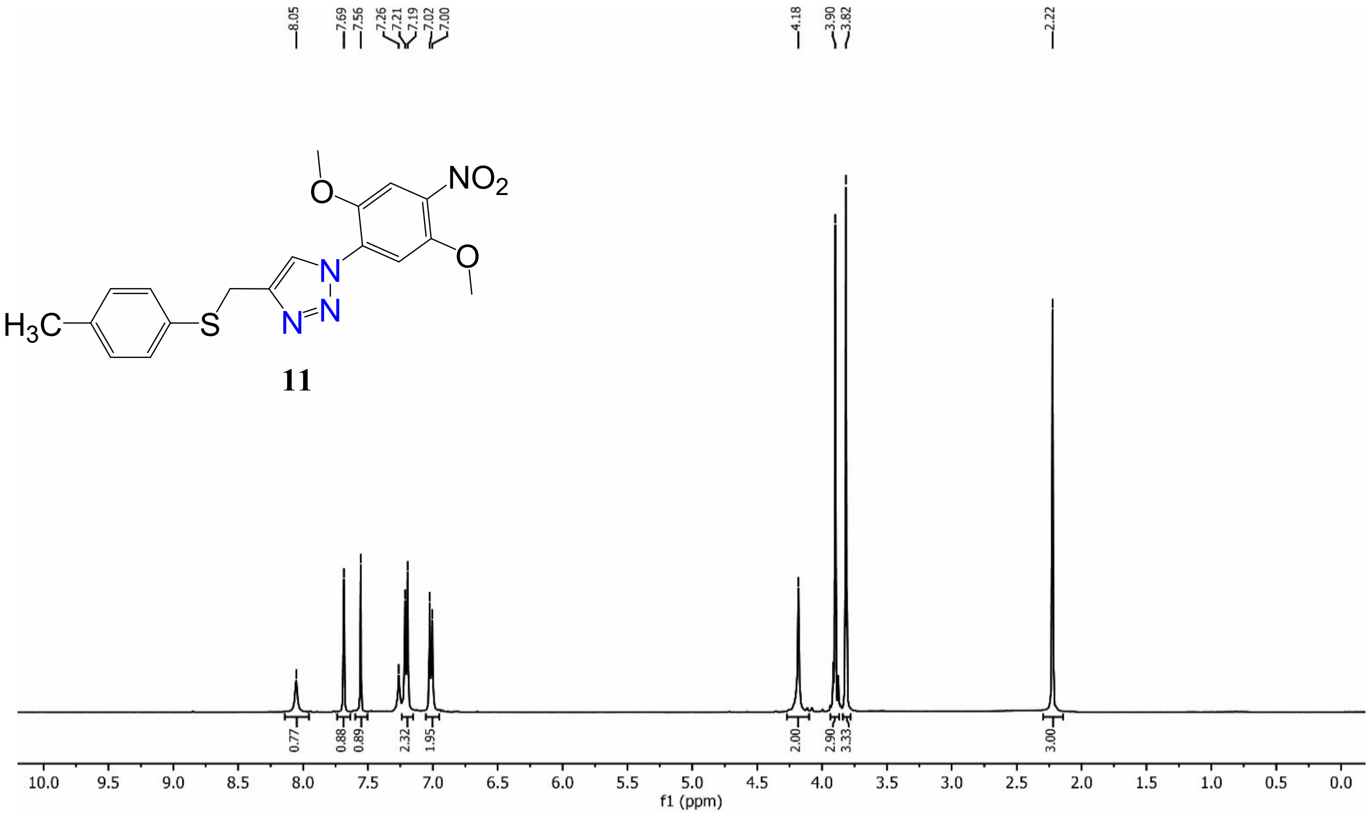

Figure S3. ^{1}H NMR spectrum of compound **11** (400 MHz, $CDCl_3$, 25 °C)……………………...

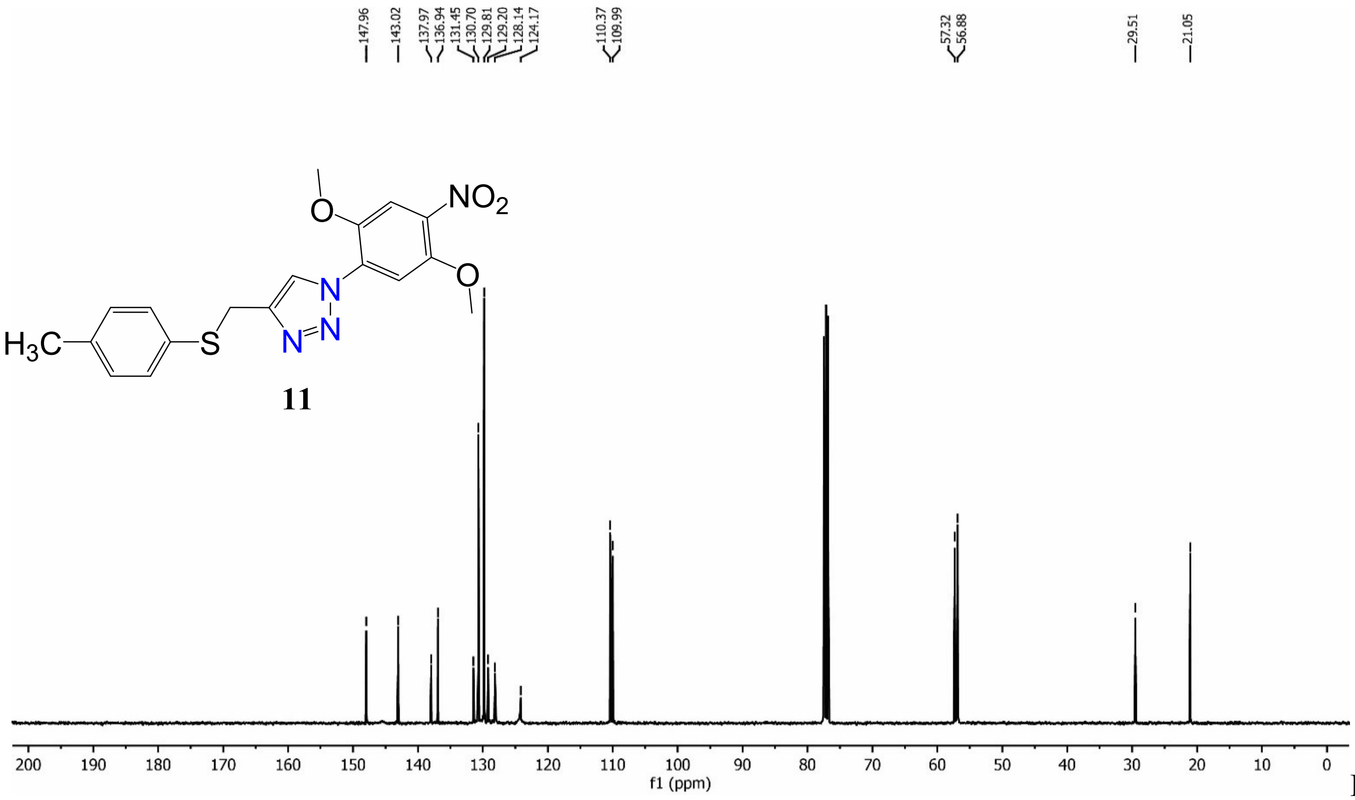

Fi

gure S4. ^{13}C NMR spectrum of compound **11** (100 MHz, $CDCl_3$, 25 °C)………………………

Compounds 12

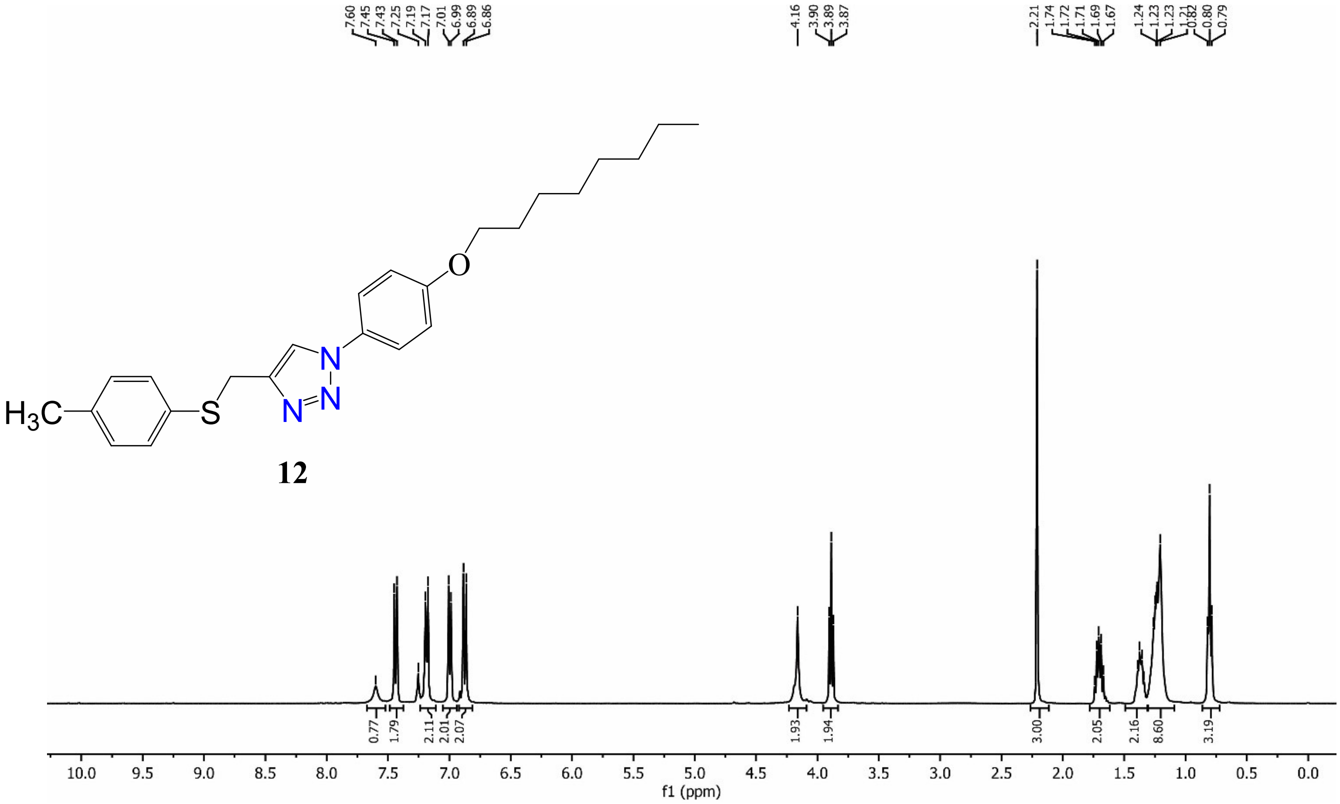

Figure S5. ^{1}H NMR spectrum of compound **12** (100 MHz, $CDCl_3$, 25 °C)………………………...

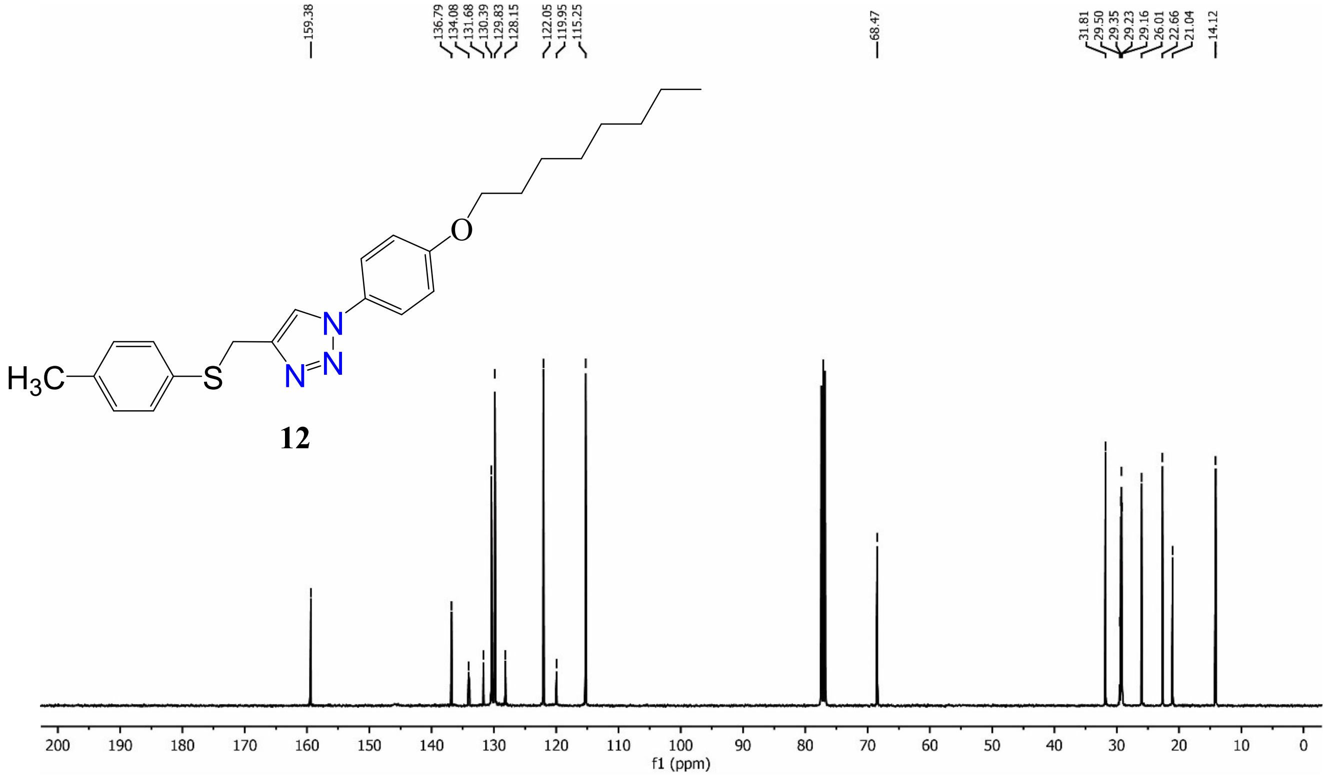

Figure S6. ^{13}C NMR spectrum of compound **12** (400 MHz, $CDCl_3$, 25 °C)……………………….

Compound 13

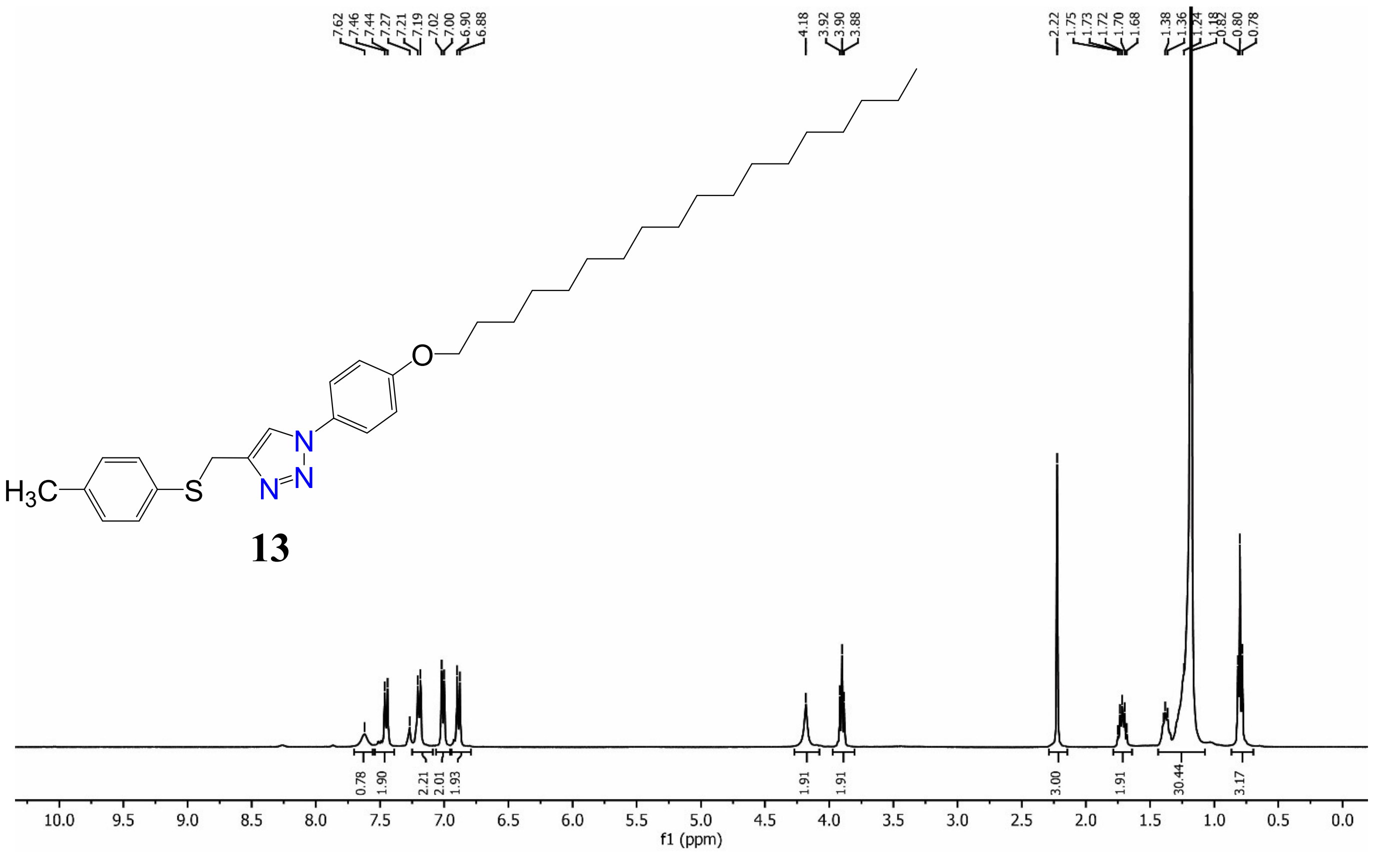

Figure S7. ^{1}H NMR spectrum of compound **13** (400 MHz, $CDCl_3$, 25 °C)…………………………...

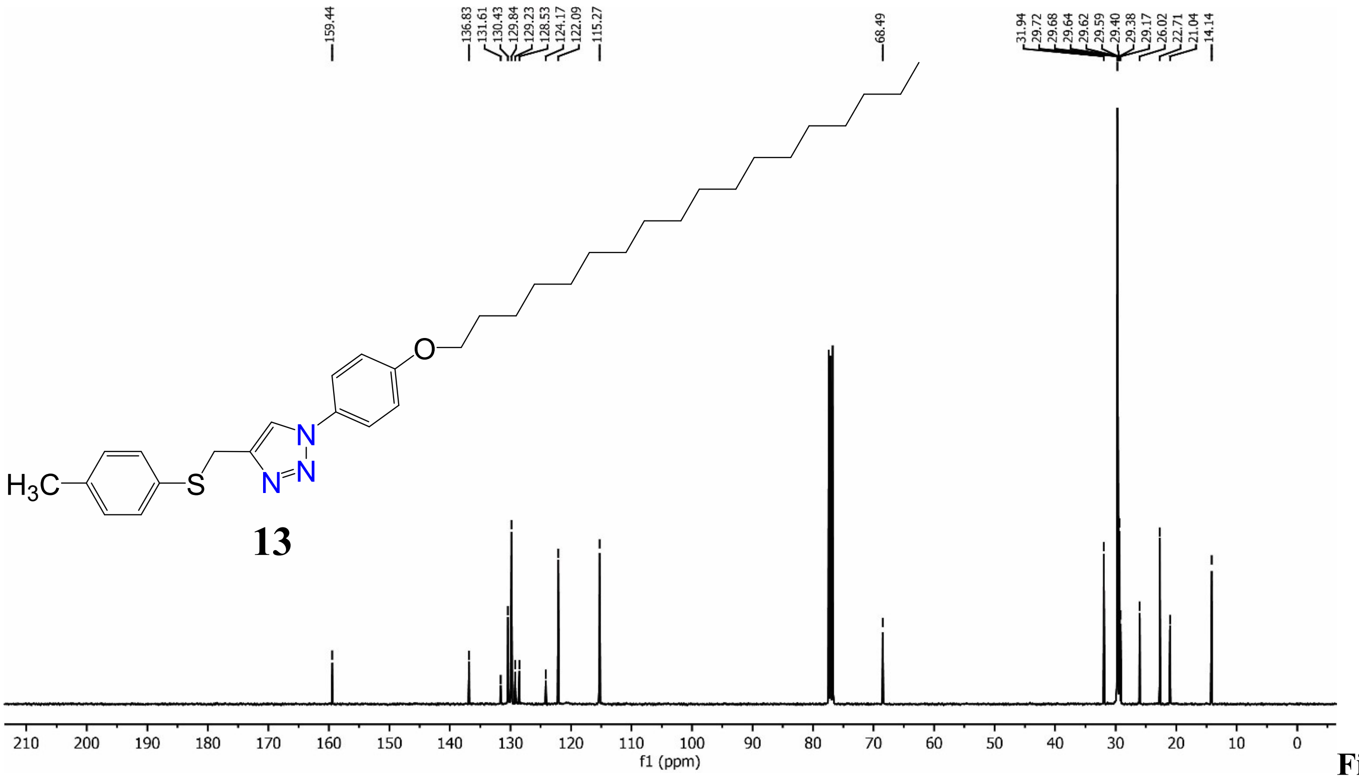

Fig ure S8. ^{13}C NMR spectrum of compound **13** (100 MHz, $CDCl_3$, 25 °C)………………………..

Compound 14

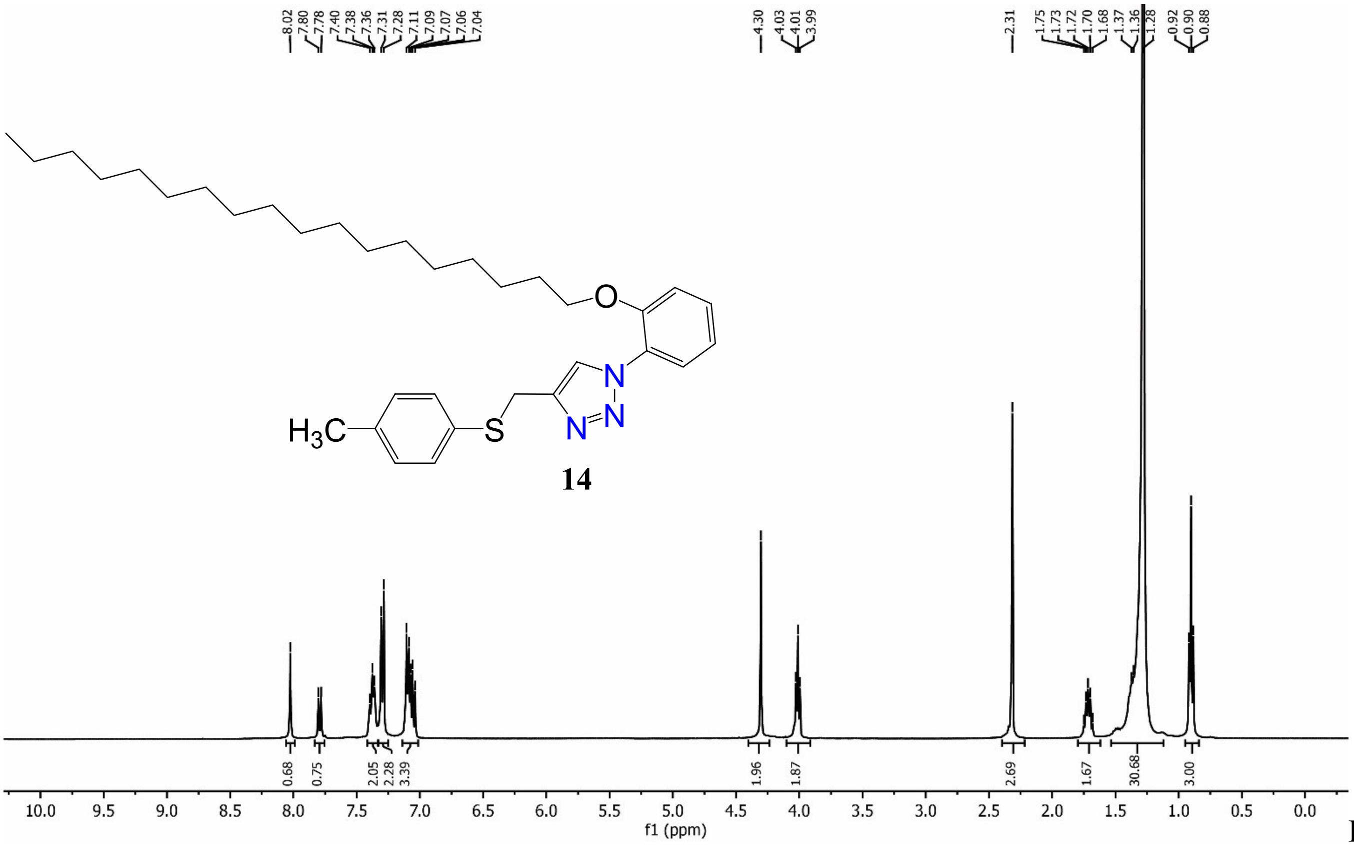

Fi

gure S9. ^{1}H NMR spectrum of compound **14** (400 MHz, $CDCl_3$, 25 °C)……………………….....

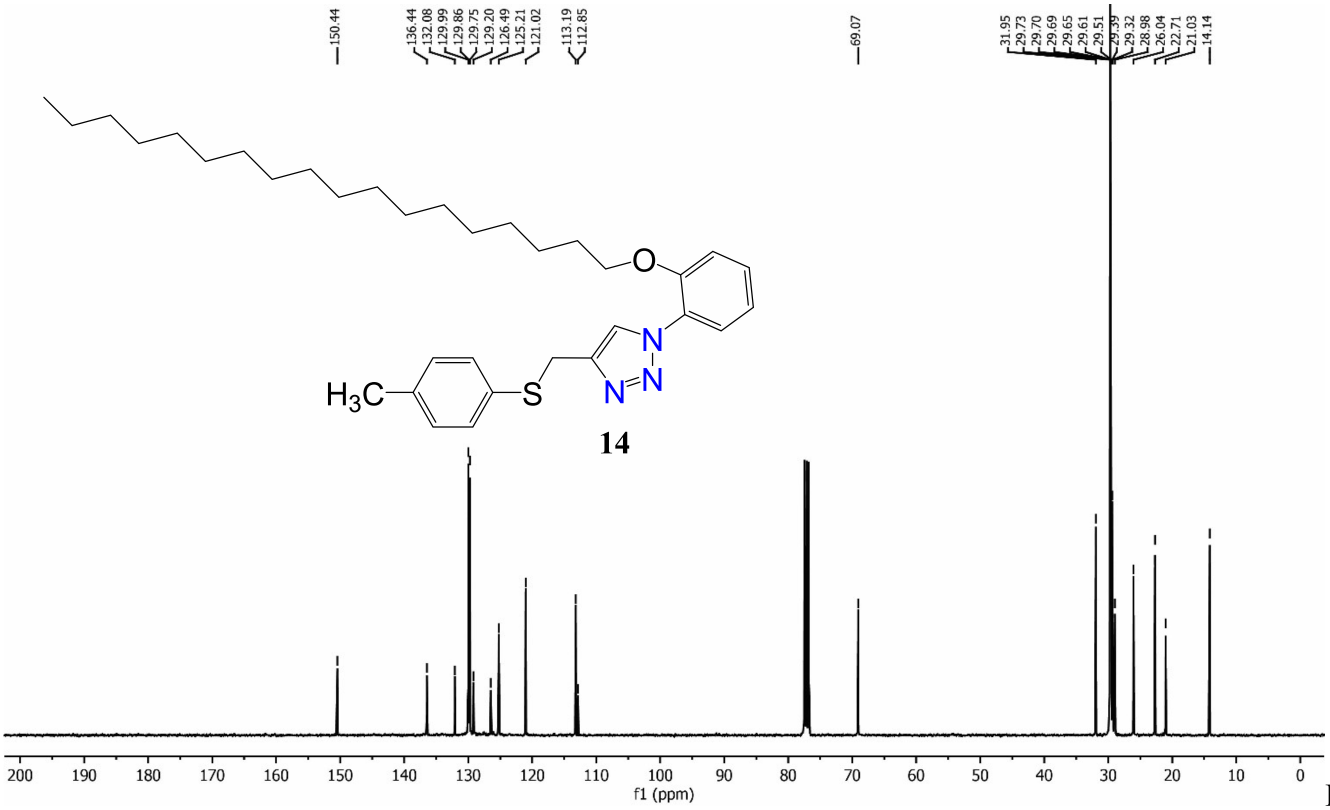

Figure S10. ^{13}C NMR spectrum of compound **14** (100 MHz, $CDCl_3$, 25 °C)……………………….

Compound 15

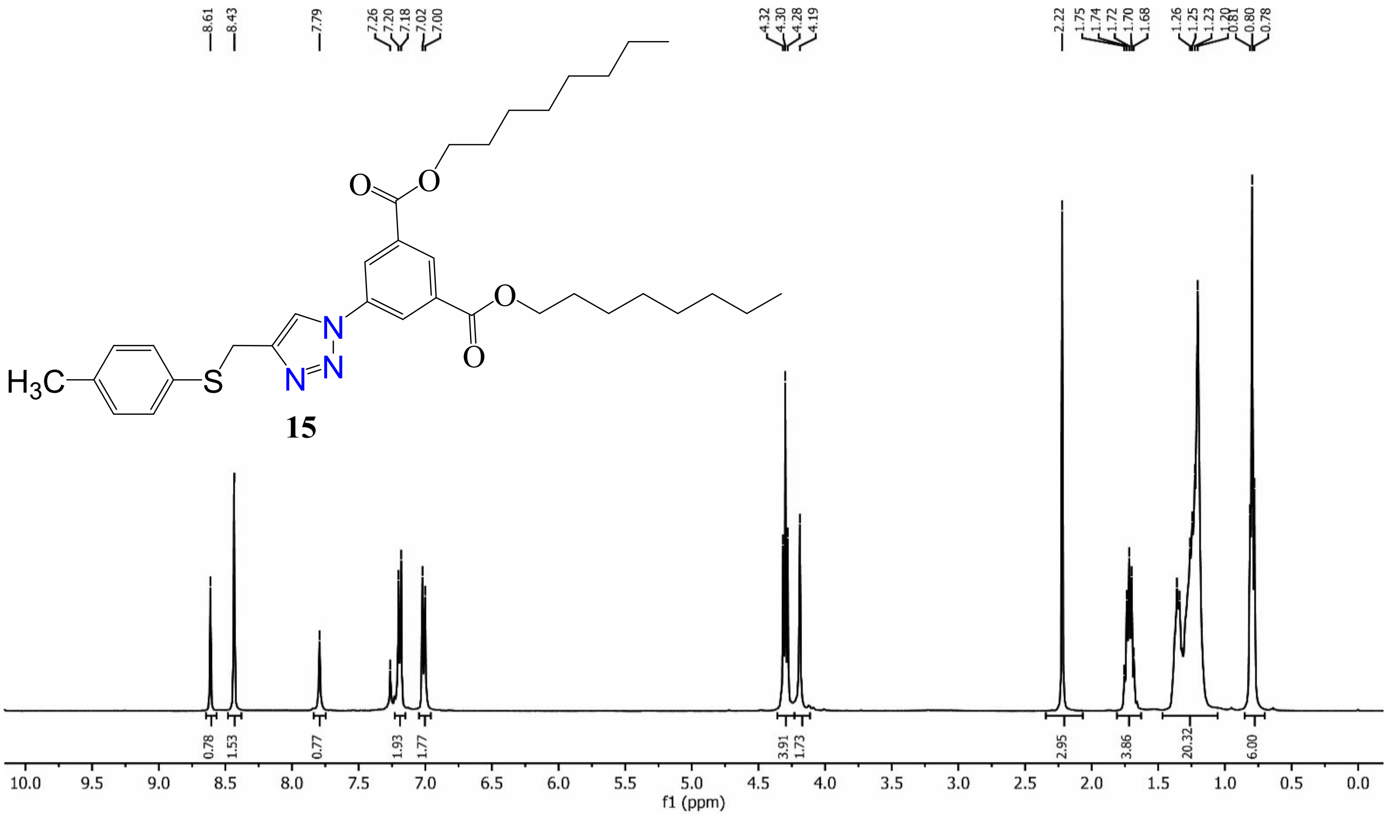

Figure S11. ^{1}H NMR spectrum of compound **15** (400 MHz, $CDCl_3$, 25 °C)……………………….

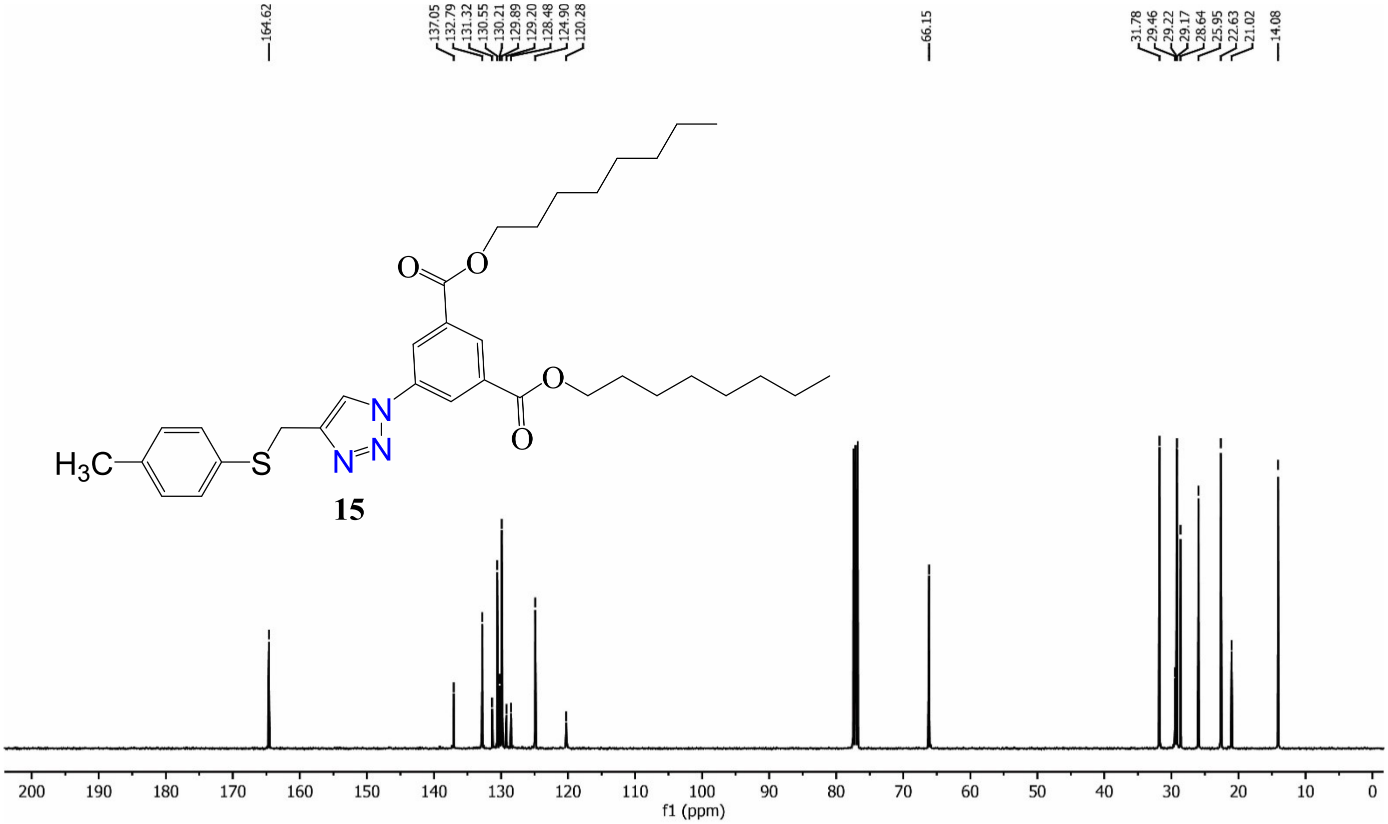

Figure S12. ^{13}C NMR spectrum of compound **15** (100 MHz, $CDCl_3$, 25 °C)………………………

Compound 16

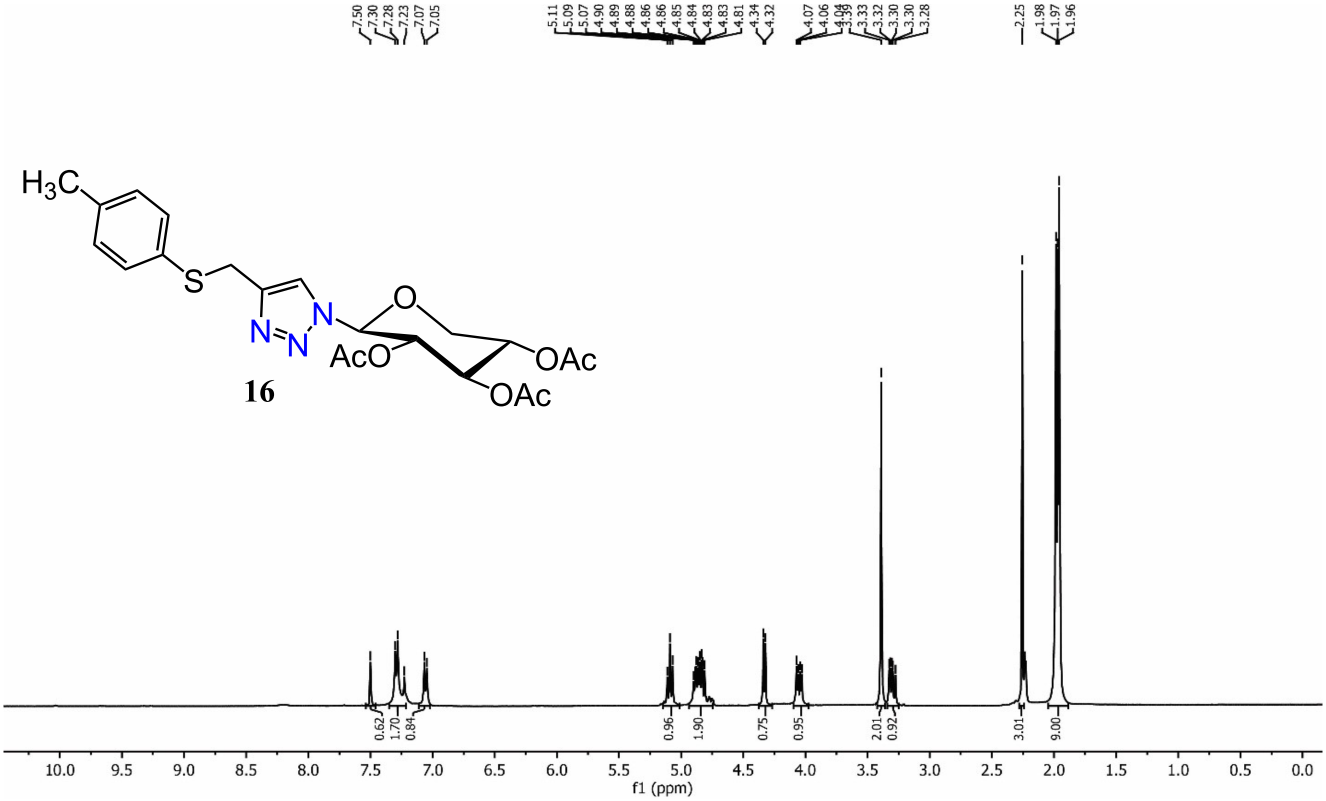

Figure S13. ^{1}H NMR spectrum of compound **16** (400 MHz, $CDCl_3$, 25 °C)……………………..

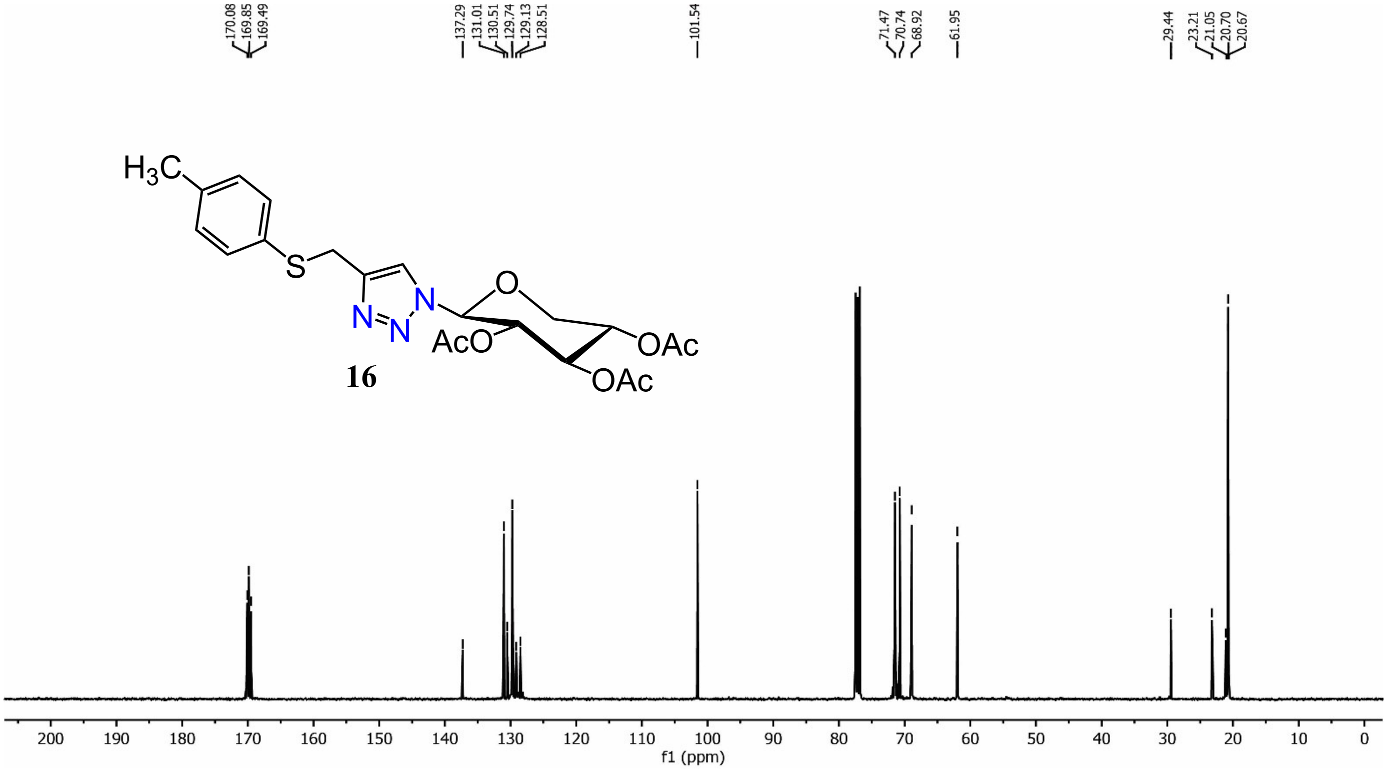

Figure S14. ^{13}C NMR spectrum of compound **16** (100 MHz, $CDCl_3$, 25 °C)……………………..

Compounds 17

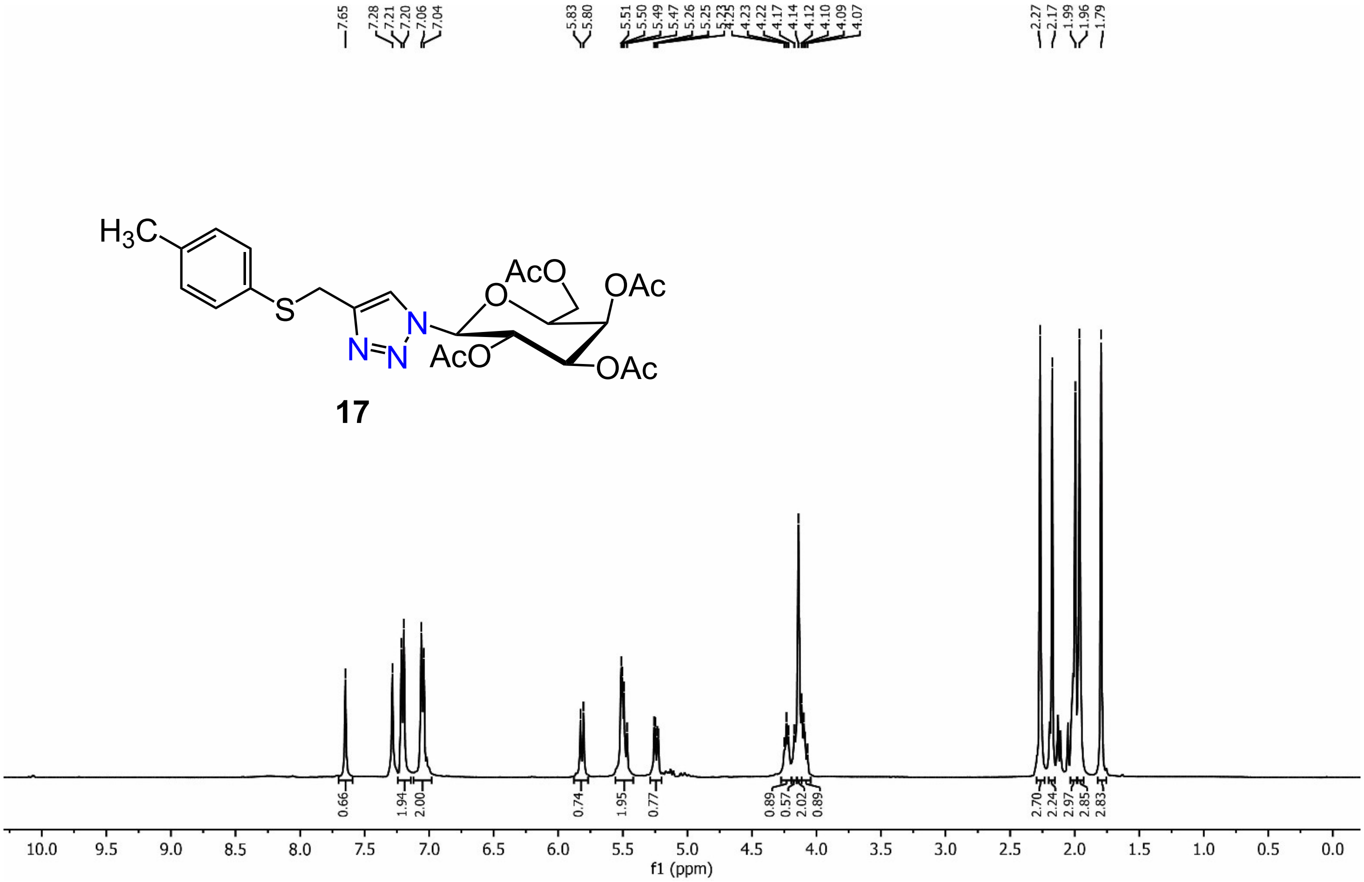

Figure S15. ^{1}H NMR spectrum of compound **17** (400 MHz, $CDCl_3$, 25 °C)……………………...

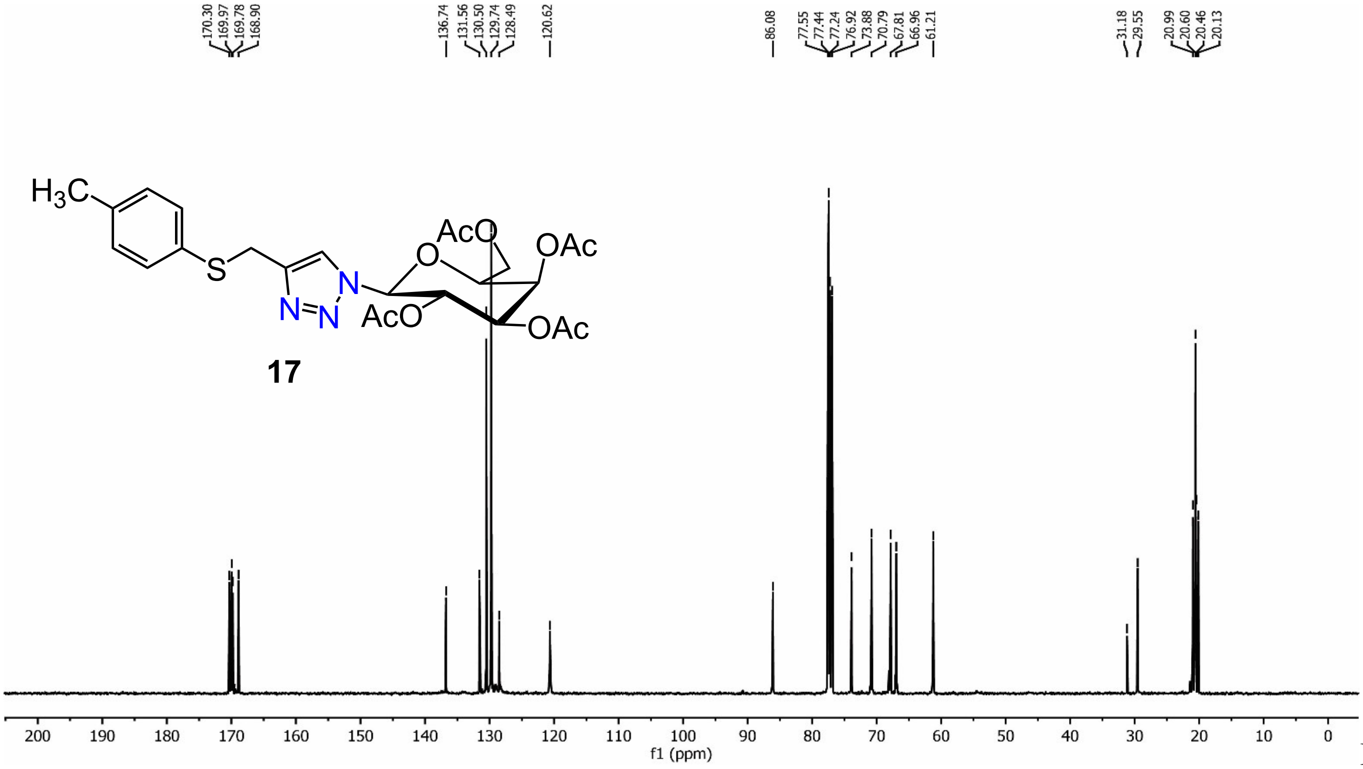

F

igure S16. ^{13}C NMR spectrum of compound **17** (100 MHz, $CDCl_3$, 25 °C)………………………
